# Transversely isotropic hyperelastic laws for 2D FEM modeling of human thoracic spine ligaments

**DOI:** 10.1038/s41598-025-23016-9

**Published:** 2025-11-11

**Authors:** Tomasz Wiczenbach, Radosław Wolny, Agnieszka Sabik, Lukasz Pachocki, Edyta Spodnik, Wojciech Witkowski

**Affiliations:** 1 Department of Mechanics of Materials and Structures, Faculty of Civil and Environmental Engineering Gdańsk University of Technology, Narutowicza, 11/12, Gdańsk, 80-233 Poland; 2 Division of Anatomy and Neurobiology, Department of Anatomy, Medical University of Gdańsk, Gdańsk, 80–210 Poland

**Keywords:** Ligament, Thoracic spine, Anisotropic, Hyperelastic, Finite element method, Engineering, Materials science, Mathematics and computing

## Abstract

A comparative analysis of three transversely isotropic hyperelastic constitutive laws is presented to characterize the mechanical behavior of spinal ligaments within finite element simulations. In each material model, the total strain energy is partitioned into ground‑matrix and fiber contributions. The ground‑matrix response was represented by three strain‑energy functions, Neo‑Hookean, Mooney‑Rivlin, and Yeoh, whereas the fiber response was captured by a fourth‑order polynomial. Constitutive parameters were calibrated against experimental uniaxial tension data from human thoracic spinal ligaments. The models were implemented via user‑defined material subroutines in Abaqus and LS‑Dyna and evaluated with finite shell elements. Performance of the afore-mentioned constitutive laws was assessed based on their ability to fit experimental data and their computational efficiency. The results indicate that, although the Yeoh model provides the best fit to the experimental data in terms of root mean square error, it tends to underestimate the fiber contribution to the overall material response, resulting in an over-stiffening effect in simulations of short-sample tensile tests. In contrast, the Neo-Hookean and Mooney-Rivlin models do not exhibit this issue.

**Supplementary Information:**

The online version contains supplementary material available at 10.1038/s41598-025-23016-9.

## Introduction

Modeling of tissues within both, the analytical and finite element method (FEM) frameworks, has significant applications in biomedical material development and implantology and is crucial in biomechanical simulations. Such simulations enable quantitative assessments of injury risk during daily activities and accidental events^1–3^, analyze surgical procedures^4^, or explore tissue healing and growth in in vitro settings^5^. Key to this field is the creation of reliable models of tissues, organs, or even entire human.

Accurate reproduction of tissue mechanics requires an appropriate constitutive law^6^. Consequently, model development starts with controlled mechanical testing^7–9^ and proceeds to mathematical formulation and parameter identification^10^.

The spinal ligaments investigated here are thin, fiber-reinforced tissues whose collagen bundles align predominantly with the principal loading direction. This pronounced anisotropy generates markedly different mechanical responses parallel versus perpendicular to the fibers^11,12^. Therefore, transversely isotropic hyperelastic models constitute the first reasonable option. There exists a wide range of such models, which differ primarily in the definition of the strain energy function. For a detailed overview and comparison, see e.g^13^. Specifically for soft tissues, various proposals can be found in the literature, including models that decompose the energy into separate contributions from the isotropic matrix, reinforcing fibers, and even fiber–matrix interactions, and have already proved effective for variety of collagenous structures, like spine ligaments^14,15^, knee ligaments, tendons, arterial walls, brain and fat tissues^6,16^.

Although commercial finite element (FE) packages provide robust and efficient solvers, their native material libraries seldom include the anisotropic hyperelastic laws required for realistic biomechanical analyses. For example, Abaqus offers the Holzapfel-Gasser-Ogden model, which is suitable for arterial walls but often fails to capture the behavior of other anisotropic soft tissues. LSDyna likewise supplies only a limited set of constitutive options for such materials^17^. Consequently, user-defined material subroutines remain indispensable for reproducing the complex response of biological tissues^18,19^. These limitations underscore the need for continued development and customization of constitutive models to achieve biomechanically accurate simulations of soft tissues.

In this work, a comparative analysis is conducted on selected transversely isotropic hyperelastic constitutive laws used to characterize the mechanical behavior of spinal ligaments. Each material law partitions the total strain energy density into ground- matrix and collagen-fiber contributions. It should be emphasized that the constitutive laws under analysis are not intended to be presented as original or innovative proposals in the field of hyperelastic material modeling. The starting point for the present study is the work^15^, in which a model was originally proposed to describe the behavior of cervical spine ligaments, where the matrix behavior was represented using the Neo-Hookean (NH) function, while the fiber behavior was modeled using a fourth-degree polynomial function of fiber stretch. This model serves as a basis for further modifications, in which the matrix behavior is described using other selected energy functions, explicitly Mooney-Rivlin (MR) and Yeoh.

To enhance the generality of the analysis, the models are implemented into commercial finite element software packages used in biomechanical simulations, namely Abaqus and LS-Dyna, via user-defined subroutines. The ligaments are treated as 2-D surface structures and modelled with the use of the shell elements. Although this assumption may seem not obvious given the actual three-dimensional geometry of these tissues, such simplification is commonly adopted, particularly in large-scale numerical models, as it significantly reduces computational cost, see^20–22^. Constitutive parameters are calibrated against uniaxialtension data from human thoracic ligaments, and model performance is quantified through goodnessoffit metrics and computational efficiency. The study specifically examines whether superior curvefitting accuracy necessarily yields correspondingly accurate FE predictions for short spinal ligaments. The present analysis is confined to low strain rates and load magnitudes that do not lead to tissue failure. Future investigations will encompass higher loading scenarios, including conditions capable of inducing damage or rupture.

## Materials and methods

A detailed description of the experimental methodology and results are available in^23^. For clarity purpose here only the main issues are outlined.

### Experimental study

Thoracic spine specimens were obtained from four body donors (72–90 years, 77.8 ± 8.3 years (mean ± SD) through a body donation programme approved by the Institutional Ethics Committee of the Medical University of Gdańsk (Ordinance No. 26/2016). These donors contributed to this study under guidelines ensuring ethical handling and use for scientific purposes. All spines were free of bone or ligament diseases, with documented donor demographics.

#### Specimens’ Preparation

**Fig. 1:**
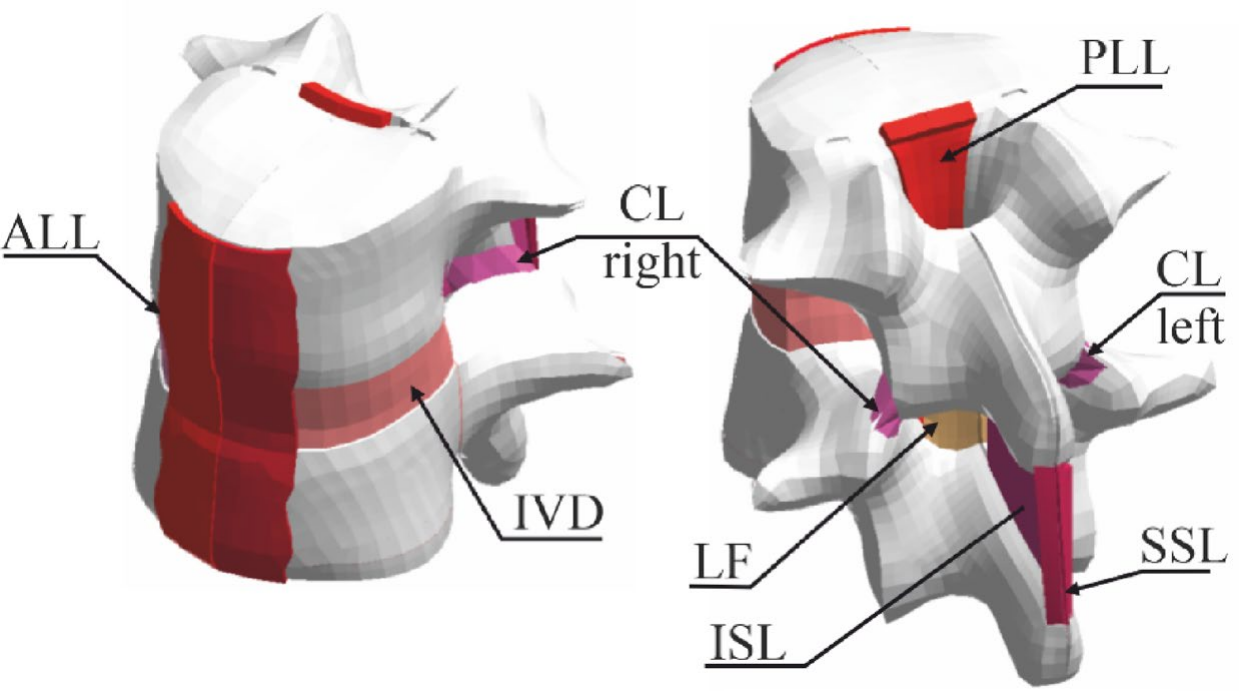
FSU model.

Each thoracic spine, harvested 24–48 h postmortem, was sectioned into functional spinal units (FSUs, see Fig. 1) spanning vertebrae Th*i*–Th*i*₊₁ (*i* = 1–11; e.g., Th2–Th3, Th4–Th5) by transecting the intervertebral discs and associated ligaments. Each FSU was then dissected into bone–ligament–bone (BLB) specimens containing the anterior longitudinal ligament (ALL), posterior longitudinal ligament (PLL), capsular ligament (CL), ligamentum flavum (LF), and the interspinous plus supraspinous ligaments (ISL + SSL). Residual intervertebral disc, muscle, and adipose tissue were removed. Specimens were wrapped in saline moistened gauze, sealed in plastic, and stored at − 20 °C until testing. In total, 17 ALL, 19 PLL, 31 CL, 17 LF, and 16 ISL + SSL samples were obtained.

#### Sample geometry measurements

**Fig. 2:**
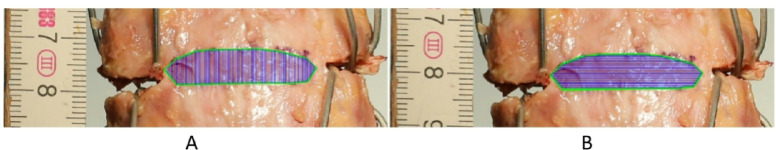
Determination of: (**A**) initial length (*L*_0_) and (**B**) width (*b*) in a CAD program.

Prior to mechanical testing, an anatomist marked the ligament-bone attachment edges with a copying pencil^7,8,24–26^. After applying a preload, photographs were taken from both sides with a reference ruler. The initial length (*L*₀) and width (*b*) was defined as the average distances between attachment points. Photographs were imported into a CAD environment (AutoCAD, Autodesk, Inc., USA). A set of vertical lines (Fig. 2A, 0.5 mm spacing) was used to determine the average length (*L*₀). Horizontal lines (Fig. 2B, 0.5 mm spacing) were used to determine the average width (*b*).

According to^27^, the cross-sectional area *A*_0_ of the samples was estimated by making use of the length-to-area ratio (*L*_v_/*A*_v_) taken after^26^. In this method the length (*L*_v_) of the ligament LF, CL, and ISL + SSL matches the measured *L*_0_. However, in the case of ALL and PLL it is the distance between the centers of adjacent vertebrae. Thus, in order to obtain *A*_0_ for ALL and PLL additional measurements of corresponding *L*_v_ distance were done. The sample thickness (*t*) was then calculated as *A*_0_ divided by the sample average width (*b*).

**Fig. 3:**
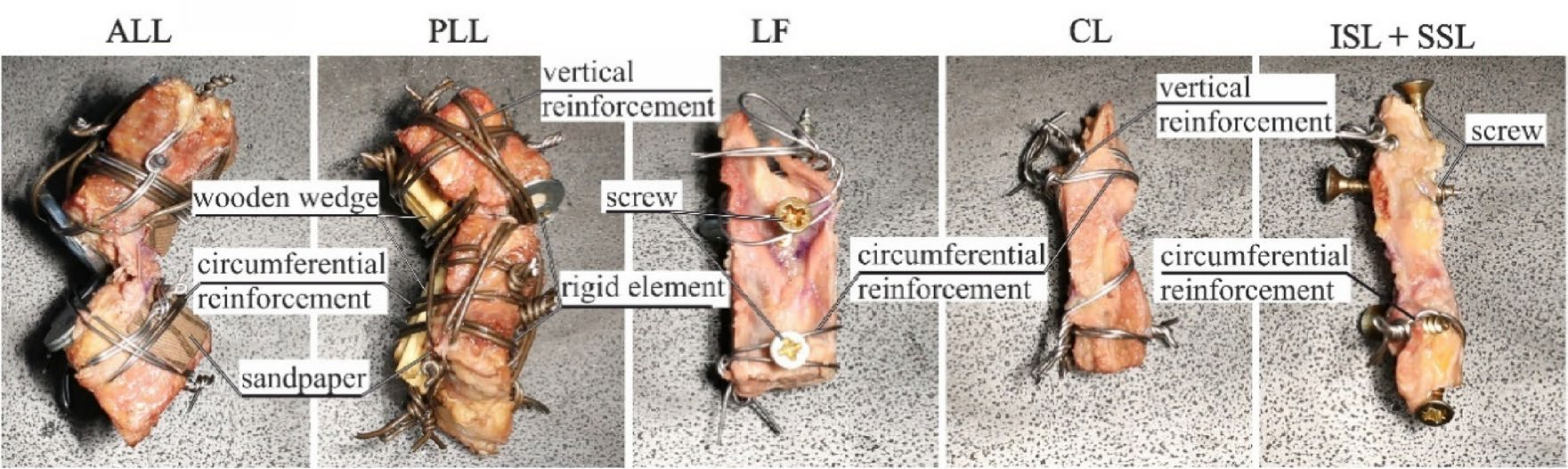
Bone parts reinforcement.

**Fig. 4:**
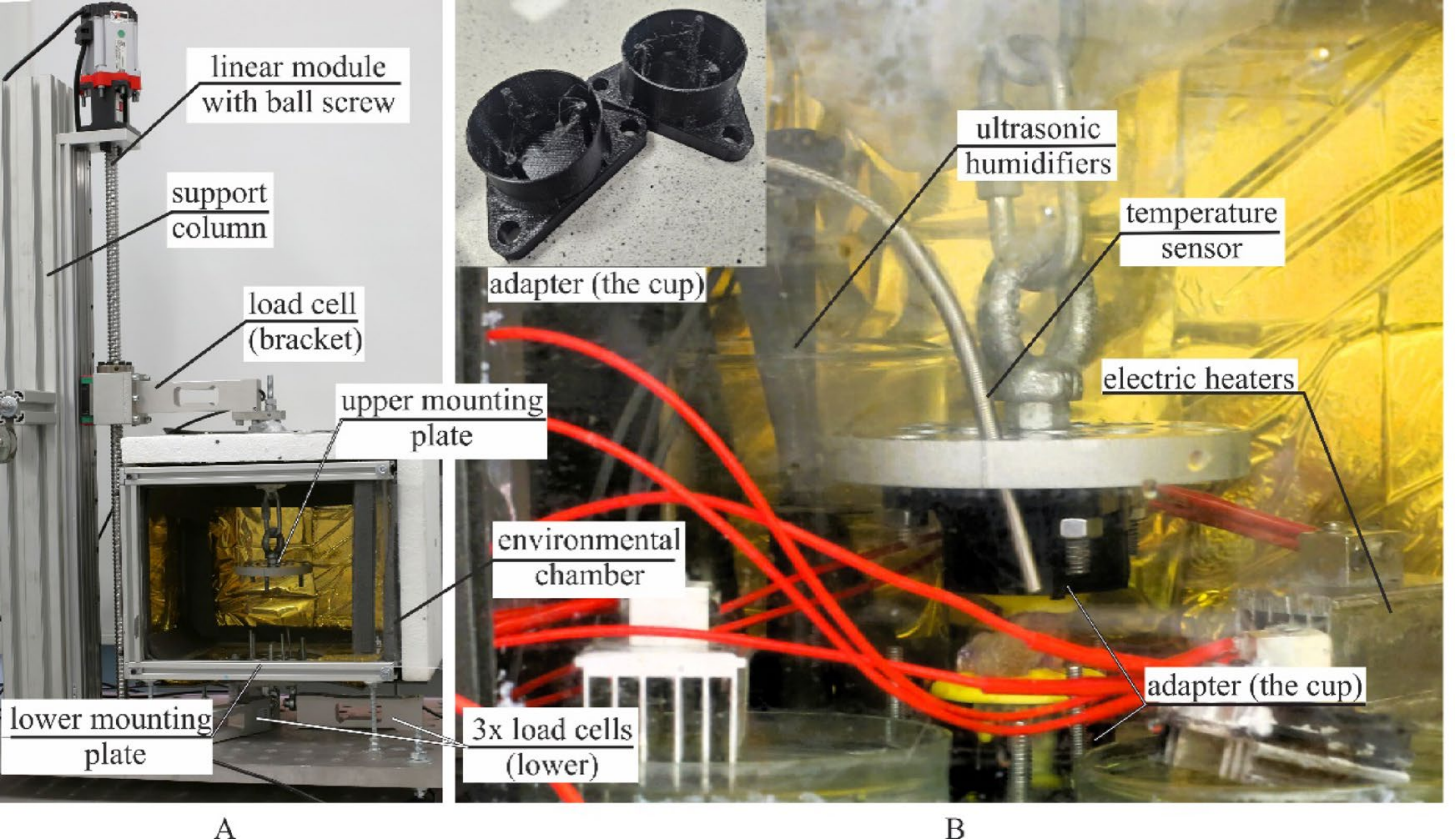
Experimental setup: (**A**) custom made machine (**B**) environmental chamber.

To minimize tissue detachment, bone sections were reinforced^8^, see Fig. 3. For ALL, a flat rigid element was placed at the site of the removed disc and wire-wrapped with the bone. On the anterior side, a compressive element was applied at the ALL attachment point and the vertebral body was circumferentially wire-wrapped. For PLL, a similar procedure was performed on the anterior side, while on the posterior side a wooden wedge with coarse sandpaper was used before wire-wrapping. The CL was secured by wire loops on both bone sides. For LF and ISL + SSL, a screw was centrally placed and circumferential reinforcement applied. To mount the samples in the testing machine, each reinforced BLB was placed into a 3D-printed adapter cups (Fig. 4B) and embedded in polymethylmethacrylate resin (Technovit 3040, Kulzer GmbH). The adapter was fastened to the machine grips using screws.

#### Experimental setup

Tensile tests were performed using a custom-designed testing machine, see Fig. 4A, (Patent application: P.443948). Displacement was controlled by an AC stepper motor (Leadshine ELM-0400LH60F-SS-400 W) with a servo controller (Leadshine ELP-RS400Z, accuracy: 0.001 mm) and a linear module (YR-HGWS60K SFU1605). Force data were recorded using three load cells (Utilcell M140, capacity up to 1500 N) mounted at the bottom. Data acquisition was managed by an HBM QuantumX MX840A system (Hottinger Baldwin Messtechnik, Germany) with Catman software.

The prepared samples were mounted in the testing machine and preloaded as described in^8^. An initial load of 5 N was applied at a strain rate of 0.5 s⁻¹, followed by a 60-second relaxation. Preconditioning consisted of 60 cycles at 10% strain and 1 Hz frequency, followed by another 60-second rest. Samples were then elongated at 0.5 s⁻¹ until failure. Tests were conducted in an environmental chamber (Fig. 4B) at 36.6 °C and ≥ 95% relative humidity maintained by electric heaters and three ultrasonic humidifiers, respectively. Conditions were monitored using a hygrometer and thermometer.

#### Data post-processing and curve averaging

The postprocessing of curves in current study followed a methodology similar to that of^23^, tailored for averaging ligament data based on stretch (*L*_0_ + *u*)/*L*_0_ and first Piola-Kirchhoff (*P*) stress *P* = *F/A*_0,_ with *F* standing for the force. Each ligament’s curve was segmented into three distinct regions: toe, linear, and subfailure, defined by four characteristic points: initial point (0,0), first transition (I), second transition (II), and failure (maximum stress), consistent with previous works by^23,28^. To determine the I and II transition points, the inflection point of the curve was first identified (difference quotient). Inflection points were identified by computing the discrete second derivative with a central finite difference scheme and linearly interpolating the zero of that derivative within the experimental data. Subsequently, two adjacent points around the inflection points were employed to establish a linear approximation. The upper and lower bounds of the linear region were then determined by locating the farthest points from the inflection point where the absolute error between the original data and the linear fit remained below 1%. The longest linear region thus formed was considered the final, true linear region, and its boundary points were designated as transition points. This procedure was applied to all curves within each ligament type, after which the transition and failure points were averaged. Utilizing the initial point, I transition, and II transition points, a polynomial or exponential function was fitted to pass precisely through these three points. Subsequently, a quadratic function was fitted between the II transition and failure points, ensuring *C*¹ continuity at the II transition point.

### Material modeling

#### Constitutive laws

From continuum mechanics viewpoint statically loaded ligament can be described as (time independent) hyperelastic material with one family of fibers (*f*) embedded in matrix (*m*). Extensive discussion of theory and applications of such materials can be found in e.g^16^. or^29^.

Hyperelastic (isothermal) material is described by strain energy function *W* which, in general, is the continuous and differentiable function of deformation gradient **F**. Due to the objectivity requirement strain energy does not depend on the rotational part of the polar decomposition of **F** but solely on right stretch tensor **U** i.e.:

1 $$W(\mathbf{F})=W(\mathbf{U})$$

In view of relation $\mathbf{U}\mathbf{U}=\mathbf{C}$ Eq. (1) may be written as

2 $$W(\mathbf{F})=W(\mathbf{U})=W(\mathbf{C})$$

with $\mathbf{C}={\mathbf{F}^{\text{T}}}\mathbf{F}$ as the right Cauchy-Green tensor. To account for anisotropic behavior of human ligaments at given material point **X** of transverse isotropic continuum with one family of fibers the preferred orientation is given by unit vector ${\mathbf{a}_0}$, along the loading direction of the tissue. In this case strain energy (2) becomes a function of two arguments:

3 $$W=W(\mathbf{C},{\mathbf{A}_0}),A_0=a_0\otimes{a}_0$$

Due to representation theorem of isotropic scalar functions e.g^30^. the strain energy may be expressed in terms of independent strain invariants *I*_*a*_, *a* = 1,2,3 of **C** or equivalently of left Cauchy-Green tensor **B** = **FF**^T^, see e.g^29^. According to^31^ there exist also pseudo-invariants of **C** and **A**_0_. Under these assumptions strain energy function (3) may be written as:

4 $$W=W\left( {{I_1}\left( \mathbf{C} \right),{I_2}\left( \mathbf{C} \right),{I_3}\left( \mathbf{C} \right),{I_4}\left( {\mathbf{C},{\mathbf{a}_0}} \right),{I_5}\left( {\mathbf{C},{\mathbf{a}_0}} \right)} \right)$$

The invariants are given as follows:

5 $${I_1}\left( \mathbf{C} \right)={\text{tr}}(\mathbf{C}),I_2(C)=\frac{1}{2}((tr(C))^2-tr(C^2)),I_3(C)=det(c)$$

where the pseudo-invariants are defined as:

6 $${I_4}\left( {\mathbf{C},{\mathbf{a}_0}} \right)={\mathbf{a}_0} \cdot \mathbf{C}{\mathbf{a}_0},{I_5}\left( {\mathbf{C},{\mathbf{a}_0}} \right)={\mathbf{a}_0} \cdot \mathbf{C^2}{\mathbf{a}_0}$$

Pseudoinvariants- *I*_4_ and *I*_5_ describe, respectively, the stretch of the embedded fiber family and its interaction with the surrounding matrix. Subsequent sections detail the numerical implementation of the transversely isotropic model in Abaqus^32^ and Ansys LS-Dyna software^33^. To accommodate near -incompressibility, the modified deformation gradient is adopted as:

7 $$\overline {{\mathbf{F}}} ={J^{{{ - 1} \mathord{\left/ {\vphantom {{ - 1} 3}} \right. \kern-0pt} 3}}}{\mathbf{F}},J=det(F)$$

The modified right Cauchy–Green tensor is defined as

8 $$\overline {{\mathbf{C}}} ={J^{ - 1/3}}{{\mathbf{F}}^T}{J^{ - 1/3}}{\mathbf{F}}={J^{ - 2/3}}{\mathbf{C}}$$

and the corresponding modified left Cauchy–Green tensor reads

9 $$\overline {{\mathbf{B}}} =\overline {{\mathbf{F}}} {\overline {{\mathbf{F}}} ^T}$$

For soft, highly hydrated tissues, the contribution of *I*_5_ is generally regarded as negligible^34–36^, and^37^. Therefore, the modified invariants of either $\overline {{\mathbf{C}}}$ or $\overline {{\mathbf{B}}}$ and pseudo-invariant used in the reminder of the paper read:

10 $$\overline {{{I_1}}} ={J^{ - 2/3}}{I_1},\overline {{{I_2}}} ={J^{ - 4/3}}{I_2},\overline {{{I_4}}} ={J^{ - 2/3}}{I_4}$$

In this paper the tissues are assumed to be fully incompressible ($J \equiv 1$)^38–42^. Following^16^ or^29^ strain energy (4) can be put into form:

11 $$W={W_m}\left( {{{\bar {I}}_1}\left( \mathbf{C} \right),{{\bar {I}}_2}\left( \mathbf{C} \right),{{\bar {I}}_3}\left( \mathbf{C} \right)} \right)+{W_f}\left( {{{\bar {I}}_4}\left( {\mathbf{C},{\mathbf{a}_0}} \right)} \right)$$

where *W*_m_ denotes the ground matrix contribution and *W*_f_ the fiber contribution. Three matrix models are considered, NeoHookean (NH), MooneyRivlin (MR), and Yeoh, collectively expressed as

12 $${W_m}=\left\{ {\begin{array}{*{20}{c}} {{W_{NH}}={C_{10}}\left( {\overline {{{I_1}}} - 3} \right)} \\ {{W_{MR}}={C_{10}}\left( {\overline {{{I_1}}} - 3} \right)+{C_{01}}\left( {\overline {{{I_2}}} - 3} \right)} \\ {{W_{Yeoh}}={C_{10}}\left( {\overline {{{I_1}}} - 3} \right)+{C_{20}}{{\left( {\overline {{{I_1}}} - 3} \right)}^2}+{C_{30}}{{\left( {\overline {{{I_1}}} - 3} \right)}^3}} \end{array}} \right.$$

while the fiber response is represented by the fourth order polynomial of fiber stretch (P4)^15^

13 $${W_f}={W_{P4}}={C_4}{\left( {{J^{2/3}}\overline {{{I_4}}} - 1} \right)^2}+{C_5}{\left( {{J^{2/3}}\overline {{{I_4}}} - 1} \right)^4}$$

For clarity, the combined models are henceforth designated as

$$NH+P4:W={W_{NH}}+{W_{P4}}$$

$$Yeoh+P4:W={W_{Yeoh}}+{W_{P4}}$$

$$MR+P4:W=W_{MR}+W_{P4}$$

#### Curve fitting procedure

Material coefficients were determined by least‑squares fitting of the uniaxial stress–stretch response predicted by each constitutive law to the experimental data. Following Abaqus documentation^32^, the Cauchy stress is decomposed as

14 $${\mathbf{\sigma }}={\mathbf{S}} - p{\mathbf{I}}$$

where *p* is the hydrostatic pressure ($p= - {1 \mathord{\left/ {\vphantom {1 3}} \right. \kern-0pt} 3}{\mathbf{I}}:{\mathbf{\sigma }}= - {{\partial W} \mathord{\left/ {\vphantom {{\partial W} {\partial J}}} \right. \kern-0pt} {\partial J}}$) and **S** denotes deviatoric (DEV) stress. For an incompressible anisohyperelastic solid with *N* fiber families this deviator is expressed as

15 $${\mathbf{S}}=\frac{2}{J} \cdot {\text{DEV}}\left[ {\left( {\frac{{\partial W}}{{\partial \overline {{{I_1}}} }}+\overline {{{I_1}}} \frac{{\partial W}}{{\partial \overline {{{I_2}}} }}} \right)\overline {{\mathbf{B}}} - \frac{{\partial W}}{{\partial \overline {{{I_2}}} }}{{\overline {{\mathbf{B}}} }^2}} \right]+\sum\limits_{{\alpha =1}}^{N} {\sum\limits_{{\beta =1}}^{\alpha } {\frac{{\partial W}}{{\partial {{\overline {I} }_{4(\alpha \beta )}}}}} } {\text{DEV}}\left( {\overline {{{{\mathbf{a}}_\alpha }}} \overline {{{{\mathbf{a}}_\beta }}} +\overline {{{{\mathbf{a}}_\beta }}} \overline {{{{\mathbf{a}}_\alpha }}} } \right)$$

with

16 $$\overline {{{{\mathbf{a}}_\alpha }}} =\overline {{\mathbf{F}}} {{\mathbf{a}}_0}_{\alpha },\overline {{\mathbf{B}}}=\overline {{\mathbf{F}}}\overline {{\mathbf{F}}}^T$$

Using the relation between the Cauchy and first Piola Kirchhoff stress (${\mathbf{P}}=J{\mathbf{\sigma }}{{\mathbf{F}}^{ - T}}$) together with conditions of incompressibility ($J \equiv 1$, ${\lambda _2}={\lambda _3}={1 \mathord{\left/ {\vphantom {1 {\sqrt {{\lambda _1} \equiv \lambda } }}} \right. \kern-0pt} {\sqrt {{\lambda _1} \equiv \lambda } }}$) and the uniaxial stress ${\sigma _{33}}={\sigma _{22}}=0$, the axial first Piola Kirchhoff stress component (${P_{11}} \equiv P={\sigma _{11}}/\lambda$) becomes:

17 $$P=2\lambda \left( {1 - \frac{1}{{{\lambda ^3}}}} \right)\frac{{\partial W}}{{\partial \overline {{{I_1}}} }}+2\left( {1 - \frac{1}{{{\lambda ^3}}}} \right)\frac{{\partial W}}{{\partial \overline {{{I_2}}} }}+2\lambda \frac{{\partial W}}{{\partial \overline {{{I_4}}} }}$$

The above equation represents the general formula for all the material models considered. The second term vanishes in the case of NH or Yeoh law. A single dominant fiber family was assumed, reflecting ligament structure^11,12^.

The curve fitting is solved with the Generalized Reduced Gradient algorithm implemented in MS-Excel. The identification of material parameters in this study is restricted to the hyperelastic response up to the second transition point, thereby excluding the softening and damage phase near ultimate failure, which is beyond the scope of the present work. The sought material coefficients generally do not possess any physical meaning except for the *C*_10_ and *C*_01_ which are assumed to depend on the initial shear modulus as follows:

18 $${C_{10}}=\frac{\mu }{2}$$

in the case of the NH and the Yeoh model and

19 $${C_{10}}+{C_{01}}=\frac{\mu }{2}$$

in case of the MR law^29,32^. As these parameters are associated with the ground substance behavior, which is mainly composed of water, it is reasonable to assume *µ* as a small value^16^. In the present approach the value of the initial shear modulus *µ*, and consequently *C*_10_ and *C*_01_, are prescribed whereas the other coefficients are obtained by the minimization method. They are assumed to be positive, which is achieved by imposing their lower bound equal to 0.001 MPa. For each ligament and each material law studied selected values of *µ* from the range $\mu \in \left( {\sim 0.001,1 - 2} \right)$ MPa are considered, see e.g. (Sopakayang, Holzapfel 2017)^38^,, whereas *µ* = 1 MPa is usually the initial guess value. Coefficient *C*_10_ in Neo-Hookean or Yeoh model approaches then 0.5 MPa, respectively, cf. (18). In the case of the Mooney-Rivlin model the contribution of *C*_10_ and C_01_ in *µ* must be estimated additionally. As this model differs from the Neo-Hookean law by the second invariant, it is reasonable to increase the impact of this quantity in the energy value. Therefore, while fitting the experimental curves with the use of the Mooney-Rivlin law, *C*_10_ is a priori reduced and set to 0.0005 MPa. Thus, if e.g. $\mu=1$is assumed C_01_ = 0.4995 MPa, cf. (19).

For each ligament and constitutive model, the quality of the total energy function and the contributions of the matrix and fibers are evaluated. It is expected, that following conditions are satisfied:

##### C1

The combined strainenergy density *W* = *W*_*m*_+*W*_*f*_ ​ must remain free of saddle points in the mean stretch space over the explored deformation range,

##### C2

The matrix contribution must be smaller than the fiber contribution (*W*_*m*_<*W*_f_), as a dominant ground matrix contribution is considered nonphysical for collagenous ligaments.

#### Numerical implementation and simulation

The constitutive models were implemented in Abaqus/Standard and LSDYNA through Fortran user subroutines. Abaqus employed the UANISOHYPER_INV interface, which requires the strain energy potential and its first derivatives with respect to the invariants in the energy function. LSDYNA used a UMAT developed for explicit time integration, necessitating direct implementation of stress-strain relations.

**Fig. 5:**
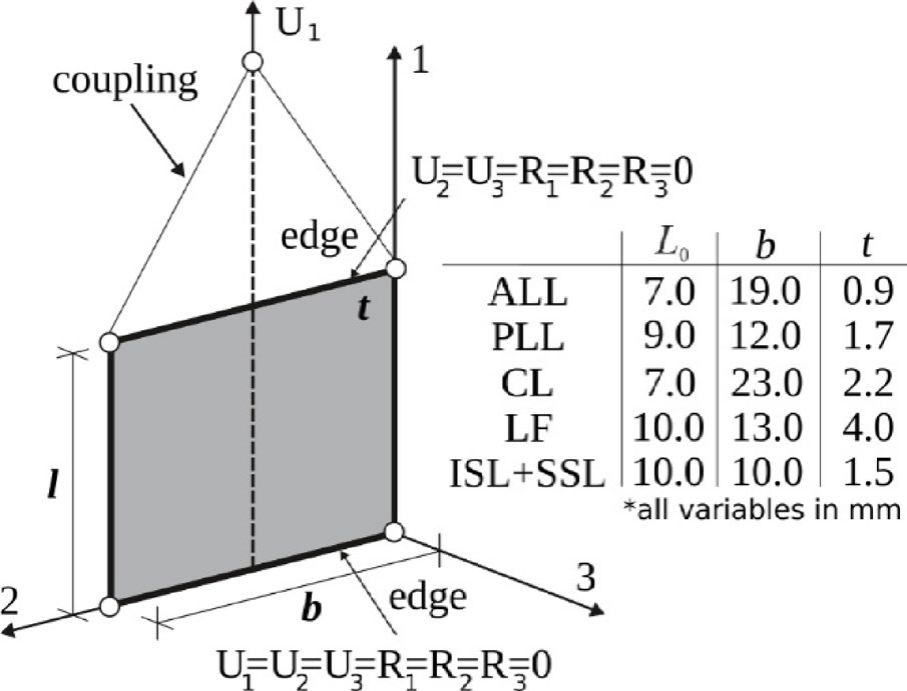
Geometrical data and boundary conditions adopted in numerical analysis.

Figure 5 depicts the geometrical data and boundary conditions adopted in the analysis. One ligament edge was fully constrained at the bone interface, whereas the opposite edge was subjected to a prescribed axial displacement by coupling all nodal degrees of freedom along that edge. Element length, width, and thickness were assigned as the mean experimental dimensions for each ligament.

Abaqus simulations were performed using the shell element with the reduced integration (S4R), assuming an approximate element size 0.5 mm. This is the only classical shell element available in this software for modeling of anisotropic hyperelastic shells. The transverse shear stiffness (${K_{TS,ij}},\,i,j=1,2$) which must be defined if the hyperelastic material is modeled is estimated as ${K_{TS,11}}={K_{TS,22}}={5 \mathord{\left/ {\vphantom {5 6}} \right. \kern-0pt} 6} \cdot \mu \cdot t$, with *t* standing for the ligament’s averaged thickness. The value of the shear modulus is assumed as.

*µ* = 1 MPa in this case. While the transverse shear effect does not arise in the uniaxial tension, more precise transverse shear stiffness definition is not required.

In LS-Dyna, the explicit analysis is based on shell element (ELFORM16) with full integration^33^. The approximate element size is maintained at 0.5 mm, consistent with the Abaqus model, ensuring a comparable spatial discretization and accuracy of the results. The transverse shear correction factor was assumed as $\kappa ={5 \mathord{\left/ {\vphantom {5 6}} \right. \kern-0pt} 6}$.

A mesh sensitivity analysis was performed to ensure that further refinement did not affect the global force–stretch response by more than 1–2%.

## Results

### Experimental results

Table 1 presents the average and SD values of initial length (*L*_0_), width (*b*), and thickness (*t*), along with the calculated cross-section (*A*_0_) for all ligaments.

**Table 1:** Ligaments geometrical properties (mean ± SD).

Ligament	*N*	L_0_, mm	b, mm	t, mm	A_0_, mm^2^
ALL	17	7.38 ± 2.4	19.42 ± 5.9	0.97 ± 0.34	17.21 ± 2.39
PLL	19	8.83 ± 1.82	12 ± 2.11	1.73 ± 0.27	20.56 ± 1.68
CL	31	6.67 ± 0.99	23.03 ± 5.67	2.2 ± 0.52	49.02 ± 7.44
LF	17	9.94 ± 2.49	12.92 ± 7.04	4.48 ± 2.13	46.1 ± 8.86
ISL + SSL	16	10.65 ± 2.4	10.36 ± 2.6	1.49 ± 0.46	14.46 ± 3.26

Table 2 presents the mean values and SD for stretch (*λ*_1_) and engineering stress (*P*_11_) at I and II transition point and failure point for all tested ligaments.

**Table 2:** Stretch (*λ*_1_) and engineering stress (*P*_11_) at I transition point, II transition point and failure point (mean value ± SD).

Ligament	I transition point		II transition point		Failure point	
	λ_1_, -	*P*_11_, MPa	λ_1_, -	*P*_11_, MPa	λ_1_, -	*P*_11_, MPa
ALL	1.16 ± 0.09	5.9 ± 3.68	1.35 ± 0.13	17.22 ± 9.01	1.62 ± 0.32	28.14 ± 12.26
PLL	1.08 ± 0.03	3.4 ± 2.11	1.17 ± 0.08	9.39 ± 5.06	1.34 ± 0.2	15.17 ± 9.55
CL	1.11 ± 0.07	1.17 ± 0.92	1.19 ± 0.1	2.82 ± 1.64	1.29 ± 0.15	4.1 ± 2.25
LF	1.19 ± 0.08	2.57 ± 1.71	1.25 ± 0.09	5.3 ± 2.72	1.3 ± 0.09	6.6 ± 3.41
ISL + SSL	1.25 ± 0.4	2.88 ± 2.11	1.32 ± 0.2	10.23 ± 8.15	1.49 ± 0.27	13.35 ± 9.35

**Fig. 6:**
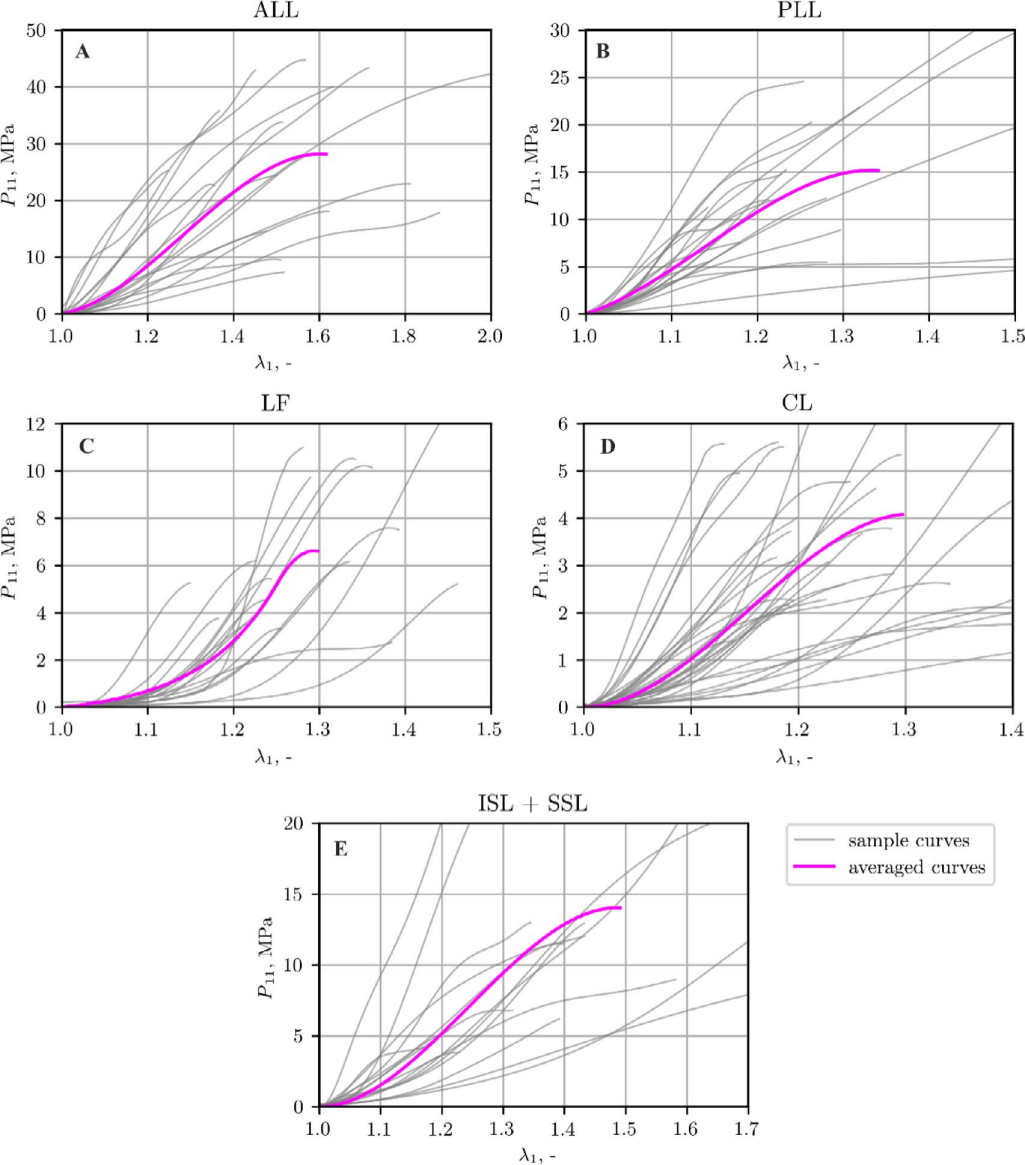
Stress-strain curves for: (**A**) ALL, (**B**) PLL, (**C**) CL, (**D**) LF, and (**E**) ISL + SSL.

Figure 6 shows experimental stress-stretch curves collected from individual tests (sample curves, grey) and averaged curve (averaged curves, magenta) for ligaments. The inter-individual variability in ligament properties (see Tables 1 and 2) was high, which is not unusual in tissue testing due to age-related and donor-specific biological factors^28^.

### Curve fitting procedure

As mentioned earlier the failure of the tissues is not considered in the study, the approximation procedure is performed up to the II transition point. The results of curve fitting procedure are presented in Supplementary Material A. From the comparison of the experimental failure stress in Fig. 6 it follows that the ligaments can be divided into two categories: stiff ligaments (ALL, PLL, ISL + SSL) and soft ligaments (CL and LF). For the sake of clarity only one stiff ligament (ALL - Figure A1-Figure A3) and one soft ligament (LF - Figure A4-Figure A6) are evaluated. In the figures the obtained material parameters together with the Root Mean Square Error (RMSE) values, stress-stretch curves, comparison of the matrix and fibers energy in the uniaxial stress state and total energy function, for different values of *µ* assumed are presented. A value of *µ* = 1 MPa was adopted as the initial guess, as it falls within the typical range reported for spinal ligaments^38,43^. To assess the influence of *µ* on RMSE and quality of the energy function, both significantly smaller value *µ* = 0.002 MPa (*C*_10_ = 0.001 MPa) and larger value *µ* = 2 MPa (*C*_10_ = 1 MPa) were considered. However, if condition C2 was not satisfied for *µ* = 1 MPa, an intermediate value *µ* = 0.2 MPa (*C*_10_ = 0.1 MPa) was employed.

### Constitutive laws in FE simulations

The validity of the examined constitutive models was next assessed in finite element tension simulations of thoracic spine ligaments. The analysis first considers the ALL and LF, representatives of the stiff and soft groups, respectively.

**Fig. 7:**
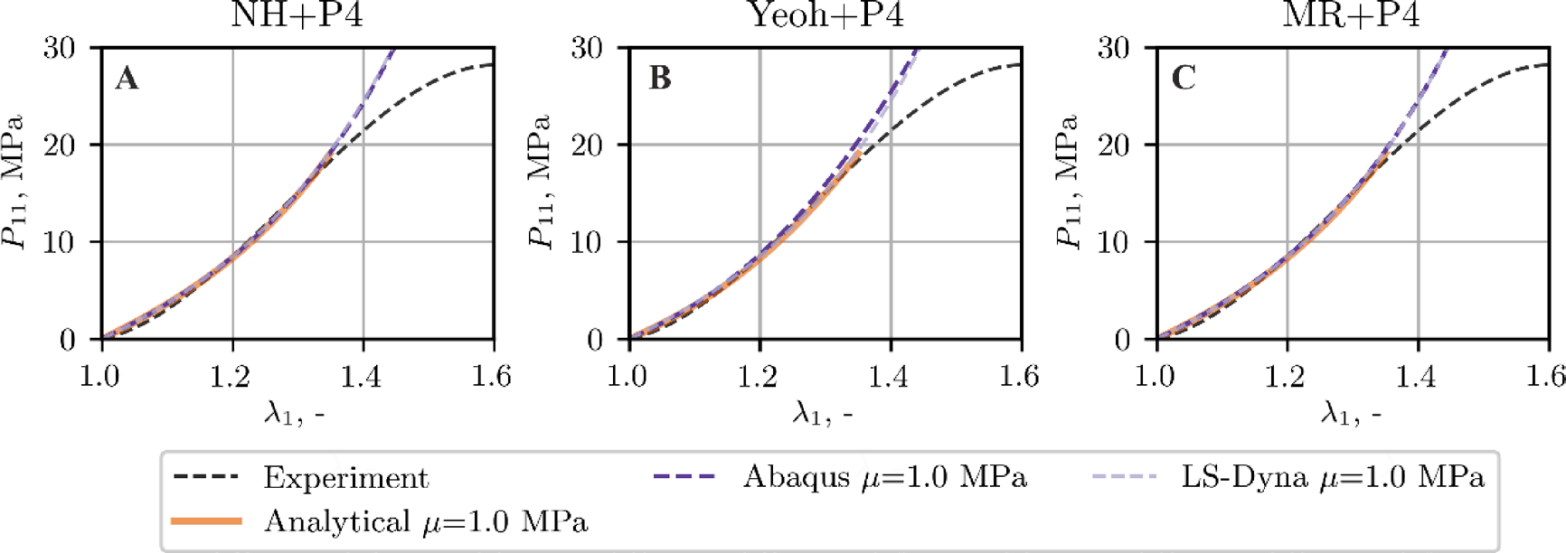
Tensile curve, ALL ligament, comparison of the experimental data, analytical data from curve fitting, and FEM results.

Owing to the fulfillment of conditions C1 and C2, see (Figure A1-Figure A3), the examination of ligament ALL is limited to only one set of material parameters, i.e. for $\mu =1.0$ MPa. Figure 7 shows the comparison between the tensile curves obtained experimentally, analytically by optimization method (as specified in Sect. 3.2) and by FEM. The FEM stress and stretch values are reported as force divided by initial cross‑sectional area and current to initial length ratio, respectively.

**Fig. 8:**
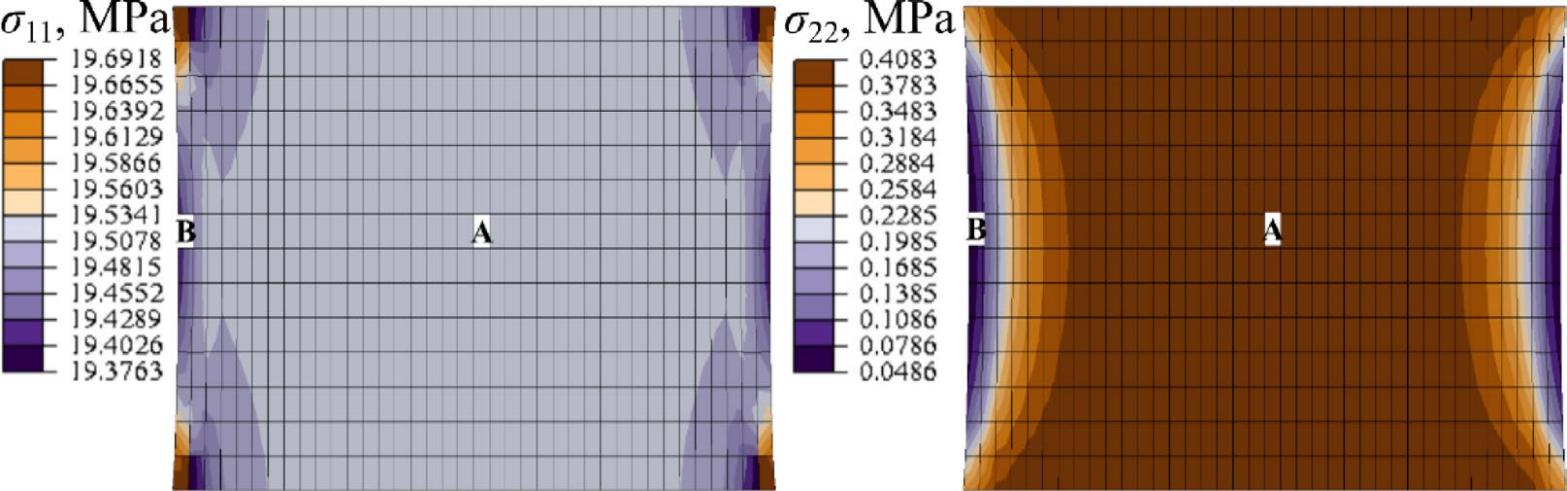
Distribution of axial (*σ*_11_) and transverse (*σ*_22_) stress at stretch $\lambda =1.3$; (NH + P4) *µ* = 1.0 MPa, ALL, Abaqus.

Figure 8, Figure B1, and Figure B2 (Supplementary Material B) illustrate the distribution of the axial (${\sigma _{11}}$) and transverse stress (${\sigma _{22}}$) in the sample obtained with different material models at stretch *λ* = 1.3.

**Fig. 9:**
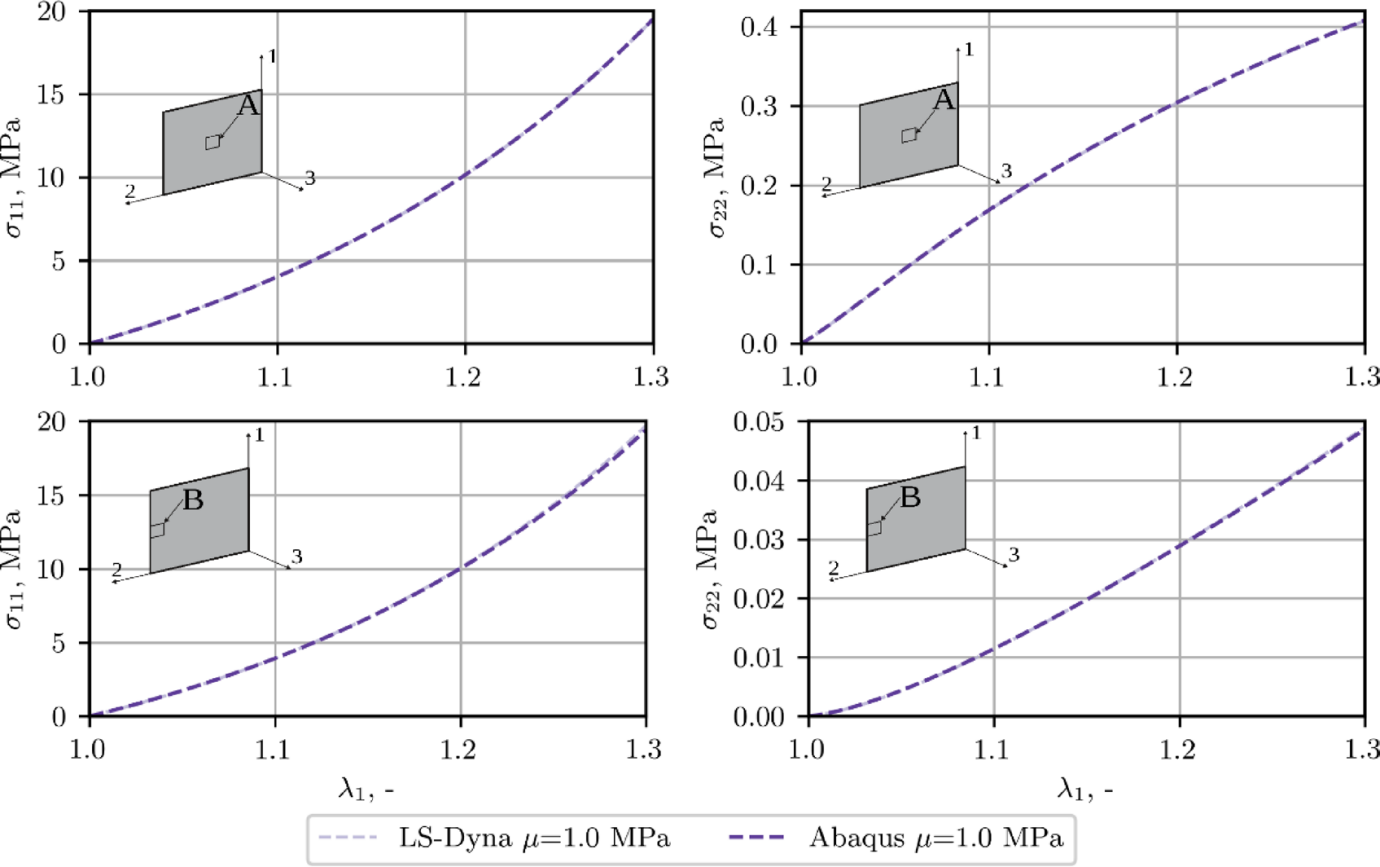
Comparison of FEM results of axial (*σ*_11_) and transverse (*σ*_22_) stress for NH + P4 (*µ* = 1.0 MPa), ALL.

For comparison, Fig. 9 shows the stress results obtained with the use of both LS-Dyna and Abaqus software, for NH + P4 (*µ* = 1.0 MPa) model in two characteristic finite elements (**A** and **B**), indicated in Fig. 8.

**Fig. 10:**
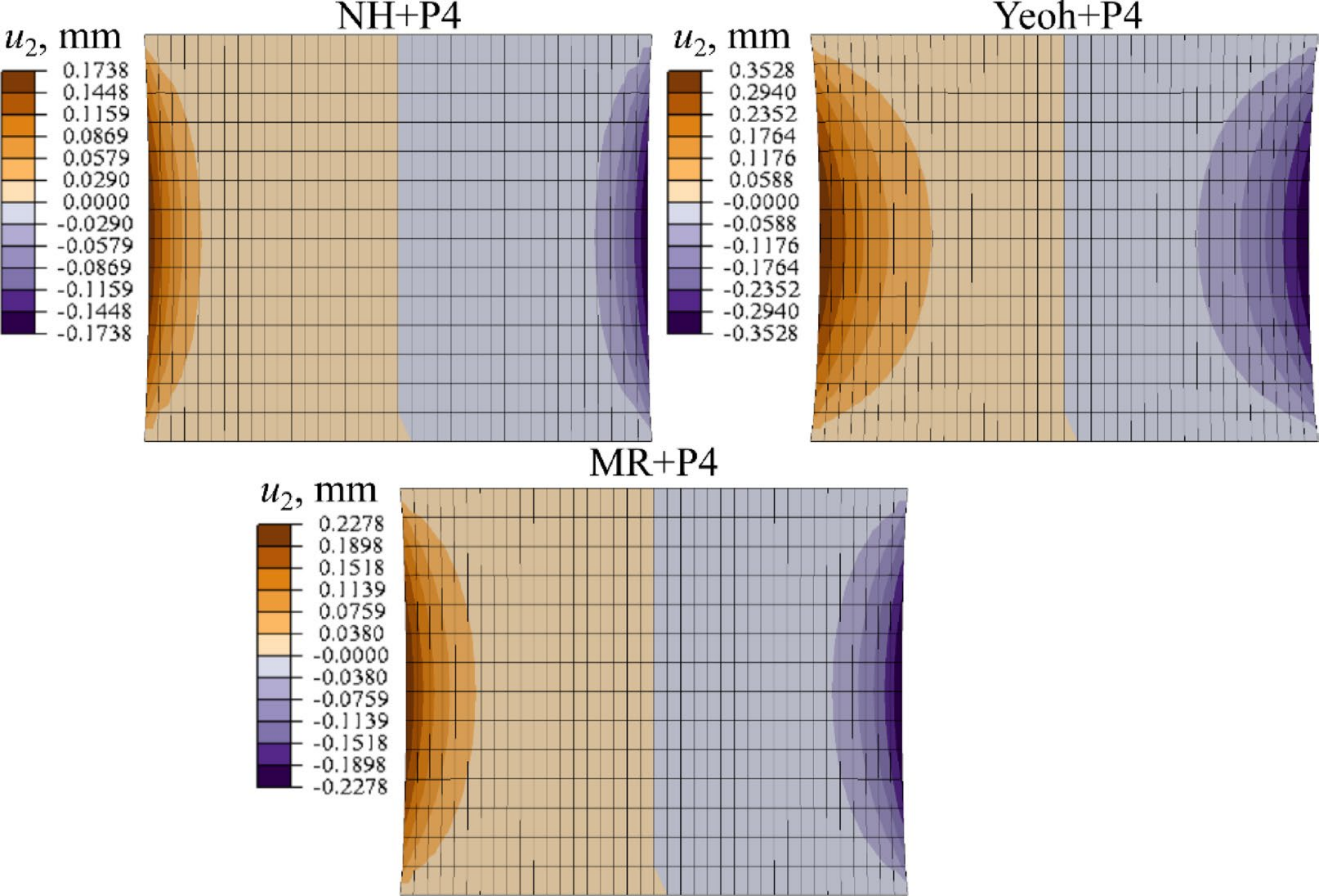
Distribution of transverse displacement at stretch $\lambda =1.3$ (*u*_1_ = 2.1 mm) for different material models (*µ* = 1.0 MPa), ALL, Abaqus.

In Fig. 10 the transverse displacement field (*u*_2_) obtained with all models is compared.

As evident from Fig. 7, all material models implemented in both FEM codes yield comparable results in terms of the global response of the analyzed sample. This consistency is supported by Fig. 9, which demonstrates close agreement in the resulting stress values. Therefore, the detailed discussion of stress distribution focuses on the Abaqus results.

**Fig. 11:**
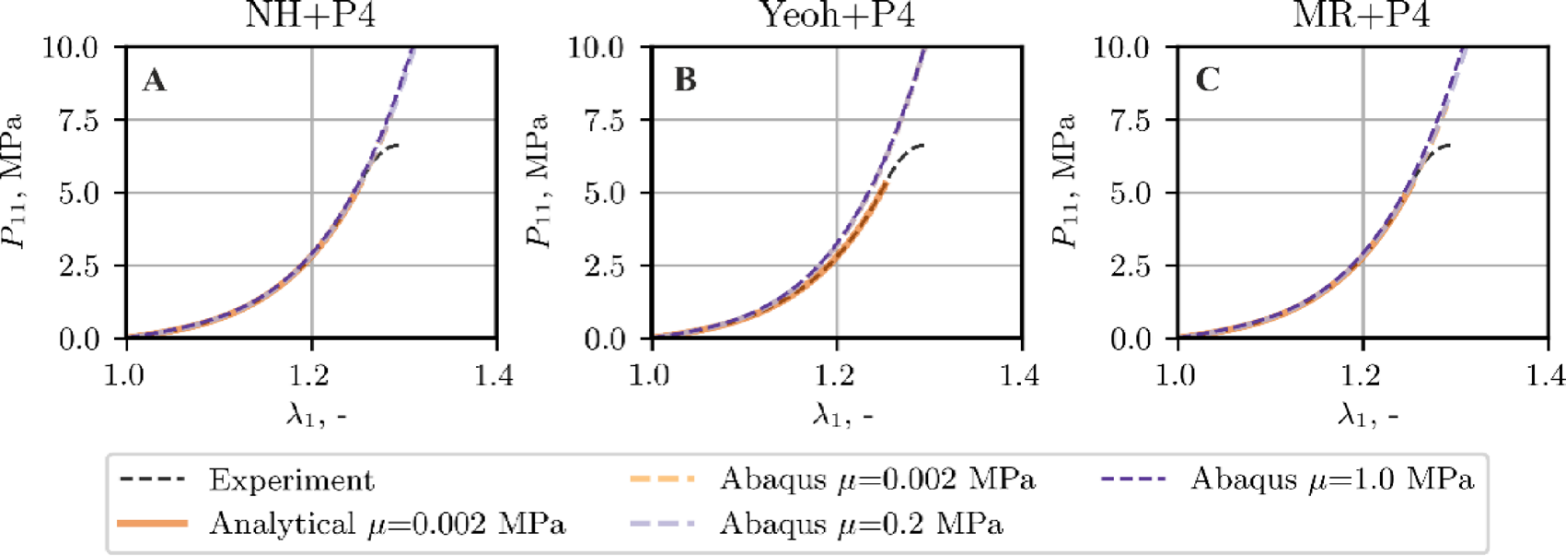
Tensile curve, LF ligament, comparison of the experimental data, analytical data from curve fitting, and Abaqus results.

**Fig. 12:**
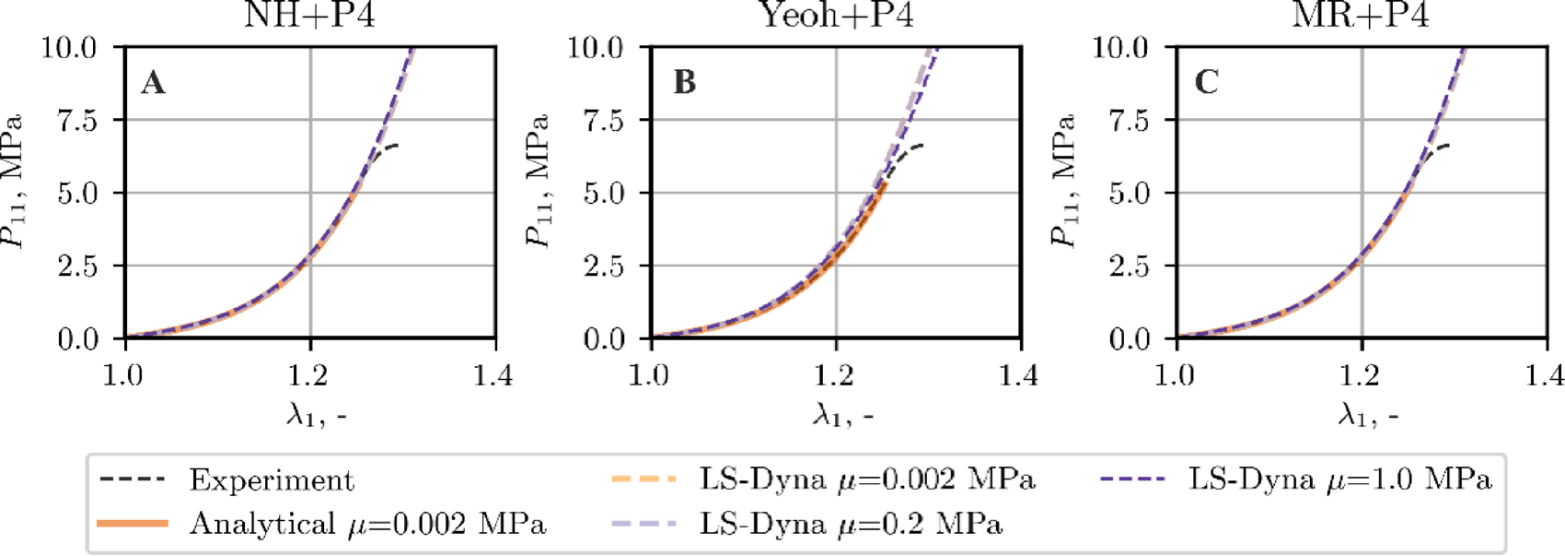
Tensile curve, LF ligament, comparison of the experimental data, analytical data from curve fitting, and LS Dyna results.

A similar study is made for a soft ligament, LF, for which, in contrast to ALL, the quality of the approximation of the stretching curve depends strongly on the chosen material model and adopted initial shear modulus *µ*, cf. Figure A4-Figure A6 (Supplementary Material A). Figures 11 and 12 illustrate the comparison between the tensile curves obtained experimentally, by curve fitting for a selected set of material parameters and computed with FEM by adopting all material models and the material parameters sets discussed in the previous section, cf. Figure A4-Figure A6 (Supplementary Material A).

**Fig. 13:**
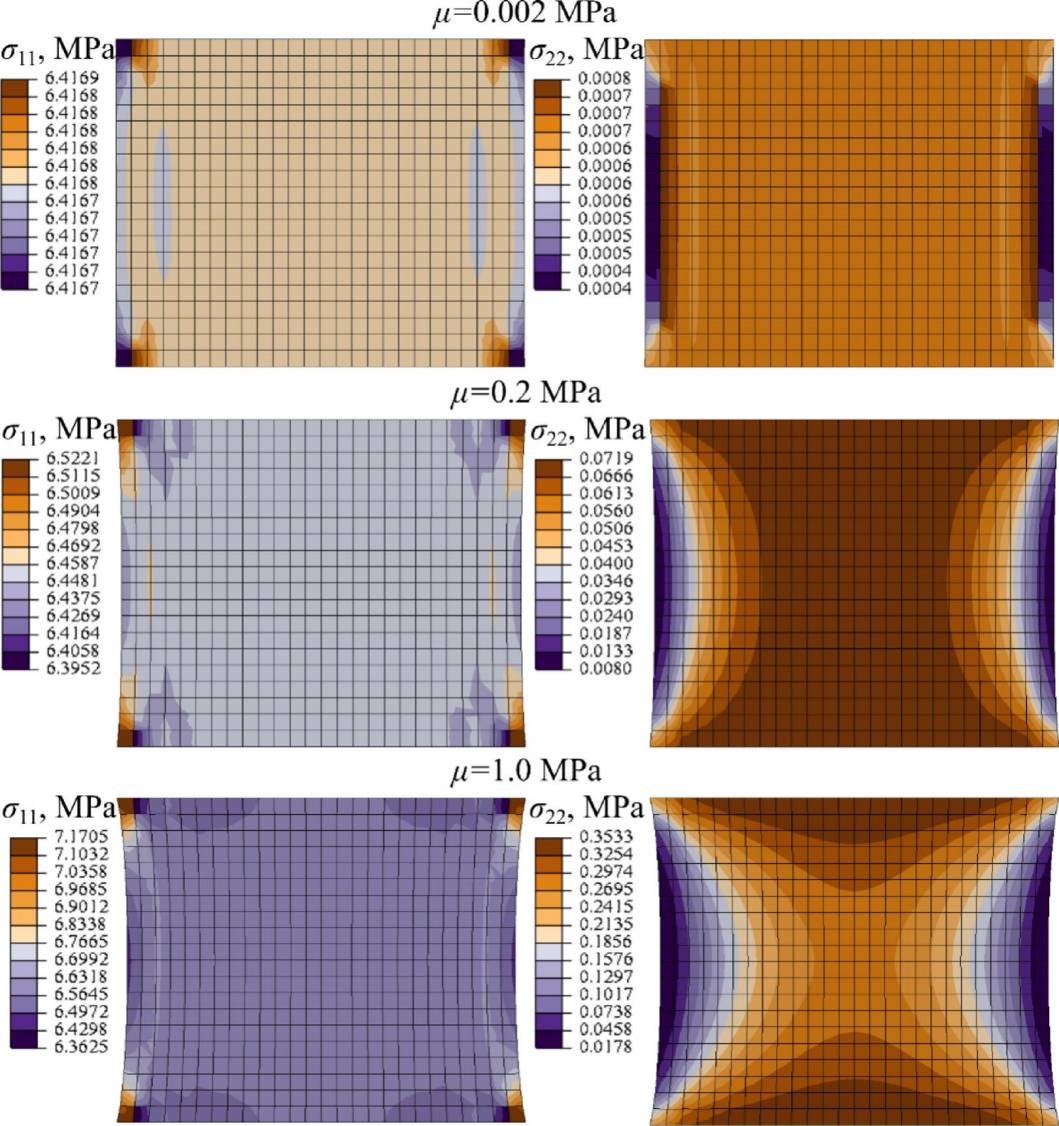
Distribution of axial (*σ*_11_) and transverse (*σ*_22_) stress at stretch *λ* = 1.25 for different material parameters sets (NH + P4), LF, Abaqus.

In Fig. 13, Figure B3 and Figure B4 (Supplementary Material B) the comparison of the axial (${\sigma _{11}}$) and transverse stress (${\sigma _{22}}$) distribution in the LF sample obtained with different material models and assuming different material parameters sets at *λ* = 1.25 is presented.

**Fig. 14:**
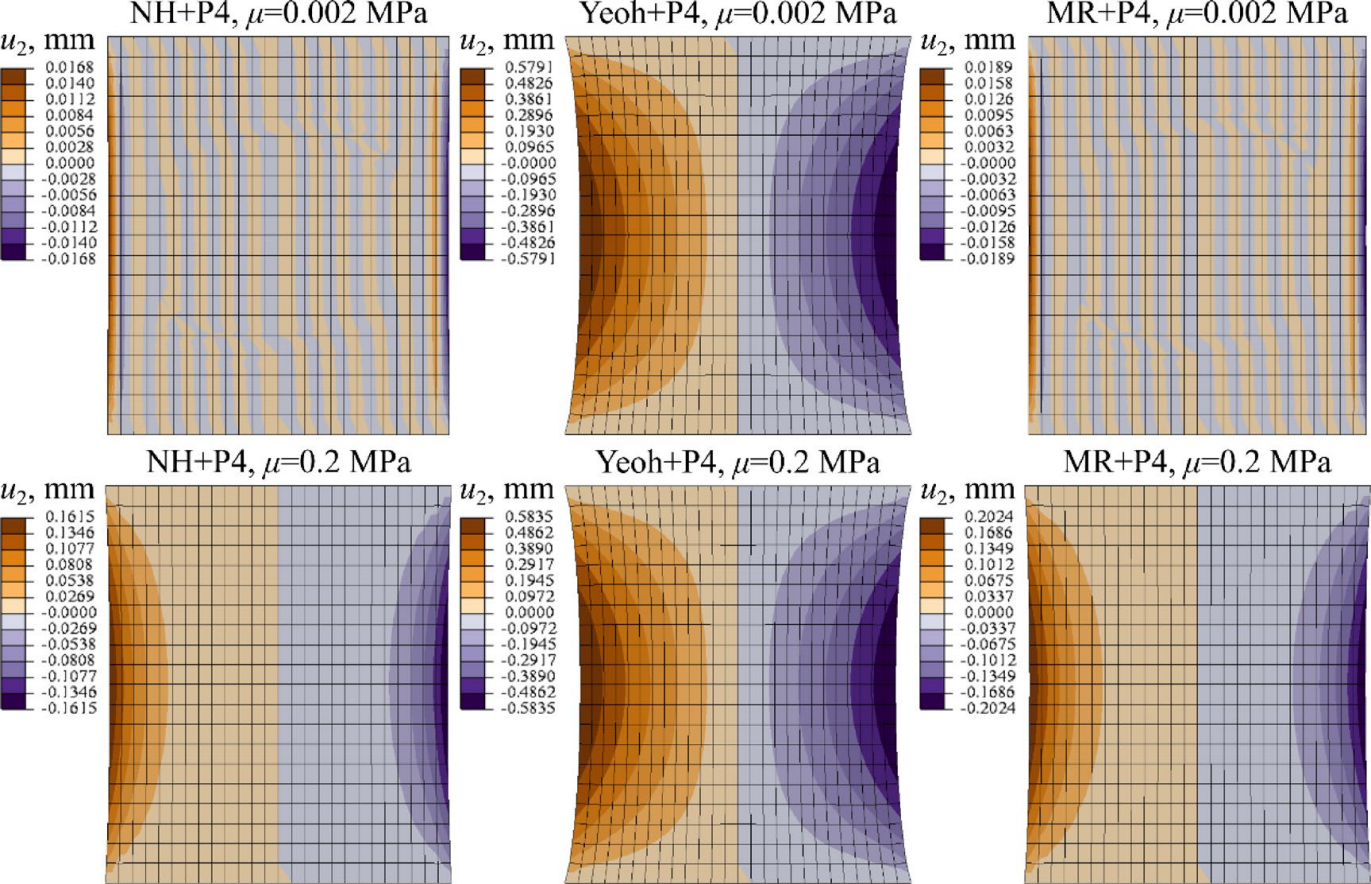
Distribution of transverse displacement at stretch(*u*_1_ = 2.5 mm) for different material models (*µ* = 0.002 MPa and *µ* = 0.2 MPa), LF, Abaqus.

Figure 14 depicts the comparison of the transverse displacement distribution (*u*_2_) in LF at *λ* = 1.25 for each material law studied. For clarity the results for selected values of shear modulus: *µ* = 0.002, *µ* = 0.2 MPa are illustrated.

Table 3 presents the material parameters of the NH + P4 model for *µ* = 1 MPa, used in simulations of the tensile response for all analyzed ligaments.

**Table 3:** Material parameters of NH + P4 model in FEM simulations.

Ligament	C_10_ [MPa]	C_4_ [MPa]	C_5_ [MPa]
ALL	0.5	3.55	0.419
PLL	0.5	4.59	1.691
LF	0.5	0.255	2.149
CL	0.5	0.673	1.152
ISL + SSL	0.5	1.846	0.626

**Fig. 15:**
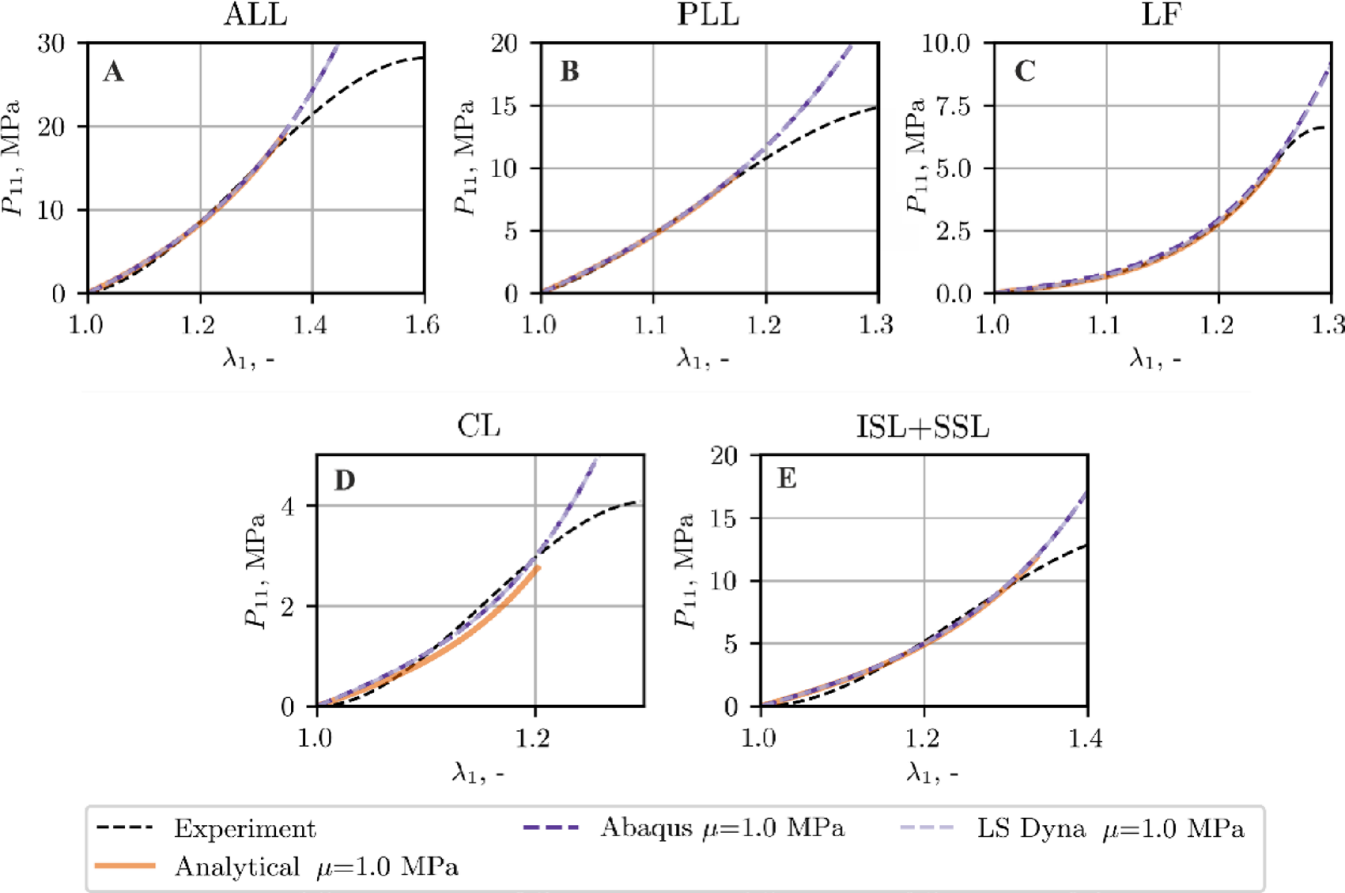
Tensile curves of the ligaments; comparison between experimental, analytical data and FEM results, NH + P4 model, *µ* = 1 MPa.

Figure 15 provides a comparative overview of experimental, analytical, and FEM-derived (Abaqus and LS-Dyna) tensile curves for each ligament.

## Discussion

### Curve fitting

The material coefficients in the considered constitutive models do not necessarily reflect physical properties but are instead calibrated to best fit experimental data. When calibration relies on a single loading condition, the resulting parameters may lack general validity. Therefore, to ensure a more comprehensive characterization of hyperelastic materials, multiple experimental tests, such as uniaxial and biaxial tension, should be employed^44,45^. However, conducting complex experiments on human cadaveric tissues is challenging due to limited availability, biological variability, and technical issues with sample preparation (see Fig. 3). Since ligaments primarily experience uniaxial loading^46^, their material behaviour is typically identified from uniaxial tensile tests^15,39^, and^47^. Nonetheless, it is still reasonable to assess the quality of the results, at least theoretically. Thus, in the present study the quality of the total energy function and the contributions of the matrix and fibers are evaluated for each ligament and constitutive model, see Sect. 2.2.2 Curve fitting procedure.

Figure A1-Figure A6 demonstrate that condition C1, is consistently met, irrespective of the constitutive law employed or the value of the shear modulus (*µ*). As indicated by the RMSE, increasing the predefined shear modulus leads to a decrease in the quality of curve fitting. This metric also reveals that the Yeoh formulation provides the best overall fit. However, condition C2 is not always fulfilled, particularly for the Yeoh model. This is mainly evident for the soft ligament LF. As shown in Figure A5, the matrix energy contribution in this case is comparable to that of fibers, and it exceeds that of fibers only in the higher stretch region for lower values of shear modulus, but for higher values of shear modulus, it exceeds fiber contribution over the entire stretch region. In contrast, for the NH + P4 and MR + P4 models, as shown in the Figure A4 and Figure A6, this effect is minor and only arises at higher shear modulus values in the lower stretch region. In the higher stretch region, the fiber contribution exceeds that of the matrix, satisfying condition C2. An incorrect balance between matrix and fiber energy terms can generate non-physical deformation modes (Canales et al. 2023). It can also complicate damage modelling, because phenomenological laws typically reduce the fiber energy to reproduce tissue softening^34,40^, and^19^.

### Constitutive laws in FE simulations

As follows from the comparison of the tensile curves presented in Figs. 7, 11 and 12, the NH + P4 and MR + P4 models provide good agreement with the experimental and analytical data, whereas the Yeoh + P4 formulation slightly overestimates the stiffness of the sample in the simulation. This discrepancy is most pronounced in the case of the soft ligament LF, regardless of the assumed shear modulus or the software used (Figs. 11 and 12).

The stiffness overestimation correlates with the pronounced transverse rigidity introduced by the Yeoh + P4 model. Based on Fig. 8, Figure B1, and Figure B2, the ratio of average transverse to longitudinal stresses (${\sigma _{22}}/{\sigma _{11}}$) at the center of the ALL ligament sample reaches approximately ~ 2% for the NH + P4 model, around ~ 4% for MR + P4, and up to ~ 10% in the case of Yeoh + P4. A comparable pattern is evident for the soft ligament LF, as shown in In Fig. 13, Figure B3 and Figure B4. For the NH + P4 and MR + P4 models, the ratio increases with the growth of the assumed shear modulus, reaching for *µ* = 1 MPa a maximum of ~ 5% for the NH + P4 and MR + P4 models, respectively. In contrast, the Yeoh + P4 model consistently yields a ratio of approximately ~ 10%, regardless of the shear modulus applied.

Transverse stiffness significantly influences the contraction of the sample. Defining the contraction ratio as the transverse-to-longitudinal displacement ratio (*u*_2_/*u*_1_), Fig. 10 shows values of ~ 8% and ~ 10% for the NH + P4 and MR + P4 models, correspondingly, and up to 16% for Yeoh + P4. For NH + P4 and MR + P4 models, this proportion depends on the assumed shear modulus, as demonstrated for the LF ligament in Fig. 14. In case of NH + P4 and MR + P4, it ranges from ~ 0.4% to 6–8%, depending on *µ*, while for Yeoh + P4 it remains as high as ~ 23%, regardless of the shear modulus.

The stiffness overestimation characteristic for Yeoh + P4, especially in case of soft ligament, can be attributed to too high contribution of the matrix in the overall load transfer mechanism (*W*_m_≈*W*_f_ or *W*_m_>*W*_f_), cf. Figure A5. This effect originates from the general structure of the Yeoh function, which is characterized by a high-degree polynomial form, rather than from specific material parameters. Consequently, the model predicts a more isotropic response, lacking directional distinction. Combined with pronounced boundary‑condition effects in short specimens, this leads to artificial stiffening. Therefore, the Yeoh + P4 model is not recommended for short‑ligament simulations.

By contrast, NH + P4 and MR + P4 preserve anisotropic mechanical behaviour and do not exhibit these limitations. The transverse stiffening produced by these material models is comparable, and they can, in the authors’ view, be used interchangeably. However, based on the previously discussed comparisons of average stress (${\sigma _{22}}/{\sigma _{11}}$) and displacement (*u*_2_/*u*_1_) ratios, the NH + P4 performs slightly better.

As shown in Figure A1 and Figure A4 the quality of curve fitting to the experimental data, measured by the RMSE, improves with decreasing values of the assumed shear modulus. However, very low values of *µ* may lead to numerical instabilities at certain levels of sample elongation. An example of such an effect is illustrated in Fig. 14. For *µ* = 0.002 MPa, characteristic checkerboard displacement patterns are observed using the NH + P4, as well as MR + P4 model, and further computations fail due to loss of convergence. Although these instabilities occur beyond the calibration range (second transition point), it is worth noting that, in damage‑capable formulations, constitutive stability at large strains is essential^19^.

Taking the above into account and since the benefits of using extremely low shear modulus values (e.g., *µ* = 0.002 MPa) compared to moderately low ones (e.g., *µ* > 0.2 MPa) are negligible, it is advisable to adopt the latter to ensure numerical robustness. Thus, in Fig. 15 the results obtained with NH + P4 model were obtained assuming *µ* = 1 MPa, see Table 3. This value together with NH + P4 model satisfies conditions C1 and C2 in case of all ligaments studied, except for the LF, for which *W*_*m*_>*W*_*f*_ is identified (Figure A4). However, this effect is observed in a very limited range of initial stretching.

As it follows from Fig. 15, the numerical results from both FEM programs show good agreement with the analytical predictions, particularly for the stiffer ligaments (ALL, PLL, ISL + SSL).

## Conclusions

This study evaluated transversely isotropic hyperelastic models for simulating the tensile behavior of human thoracic spinal ligaments using FE analysis. Three matrix models, NH, Yeoh, and MR, were combined with a fourth-order polynomial of fibers stretch (P4). Material parameters were calibrated against uniaxial tensile test data, and simulations were performed using shell elements. To enhance the general applicability of the results, the models were implemented in two commercial programs, Abaqus and LS-Dyna via user subroutines. The study examined also whether a good experimental fit necessarily translates into reliable FE performance for short ligaments under tension.

Following conclusions can be drawn:

- Among the evaluated models, the Yeoh formulation provided the best fit to the experimental data in terms of RMSE. However, this does not necessarily guarantee accurate performance in finite element simulations.
- Due to its high-degree polynomial form, the Yeoh + P4 model tends to overestimate matrix involvement in load transfer, resulting in significant transverse stiffening. Combined with boundary condition induced constraints, this results in an overall artificial stiffening of the model. Thus, the Yeoh + P4 law is not recommended for simulating the tensile behavior of short spinal ligaments.
- In contrast, the NH + P4 and MR + P4 models do not exhibit excessive transverse stiffening. Since their behavior is comparable, they may be used interchangeably. For these models, the fitting accuracy improves as the shear modulus decreases. However, excessively low shear modulus values make the material overly compliant in the transverse direction, which may lead to numerical instabilities under certain stretch conditions.
- These conclusions hold true across two independent and widely used finite element platforms, Abaqus and LS-Dyna.
- Our study demonstrates that evaluating the effectiveness of constitutive models intended for finite element implementation cannot be limited to their analytical fit to experimental data alone.

A limitation of this study is the exclusive focus on static loading conditions, with viscoelastic effects and tissue damage not being considered. These aspects will be addressed in future investigations. Additionally, the incorporation of the poroelastic behavior of biological soft tissues were not included in the analyzed models. This could offer a more physiologically accurate representation of soft tissue mechanics.

## Supplementary Information

Below is the link to the electronic supplementary material.

Supplementary Material 1

## Data Availability

The datasets used and/or analyzed during the current study are available from the corresponding author upon reasonable request. Raw data contain identifiable donor information and are therefore not publicly deposited to protect participant confidentiality.
