# Supplementary material for: Transversely isotropic hyperelastic laws for 2D FEM modeling of human thoracic spine ligaments
Source: Sci Rep. 2025 Nov 11;15:39483. doi: 10.1038/s41598-025-23016-9 (PMC12606164; doi:10.1038/s41598-025-23016-9)
Supplement: Supplementary file 1 — Supplementary Material 1 [file 41598_2025_23016_MOESM1_ESM.docx]

# Supplementary Material A

The appendix presents the detailed results of the curve fitting procedure made for stiff ligament (ALL) and soft ligament (LF). For each ligament type and each law considered following results are reported:

- the material parameters obtained via curve fitting,
- the corresponding stress-stretch curves compared with the experimental data,
- contribution of the fibers and matrix in total energy potential
- energy function in the space of principal stretches


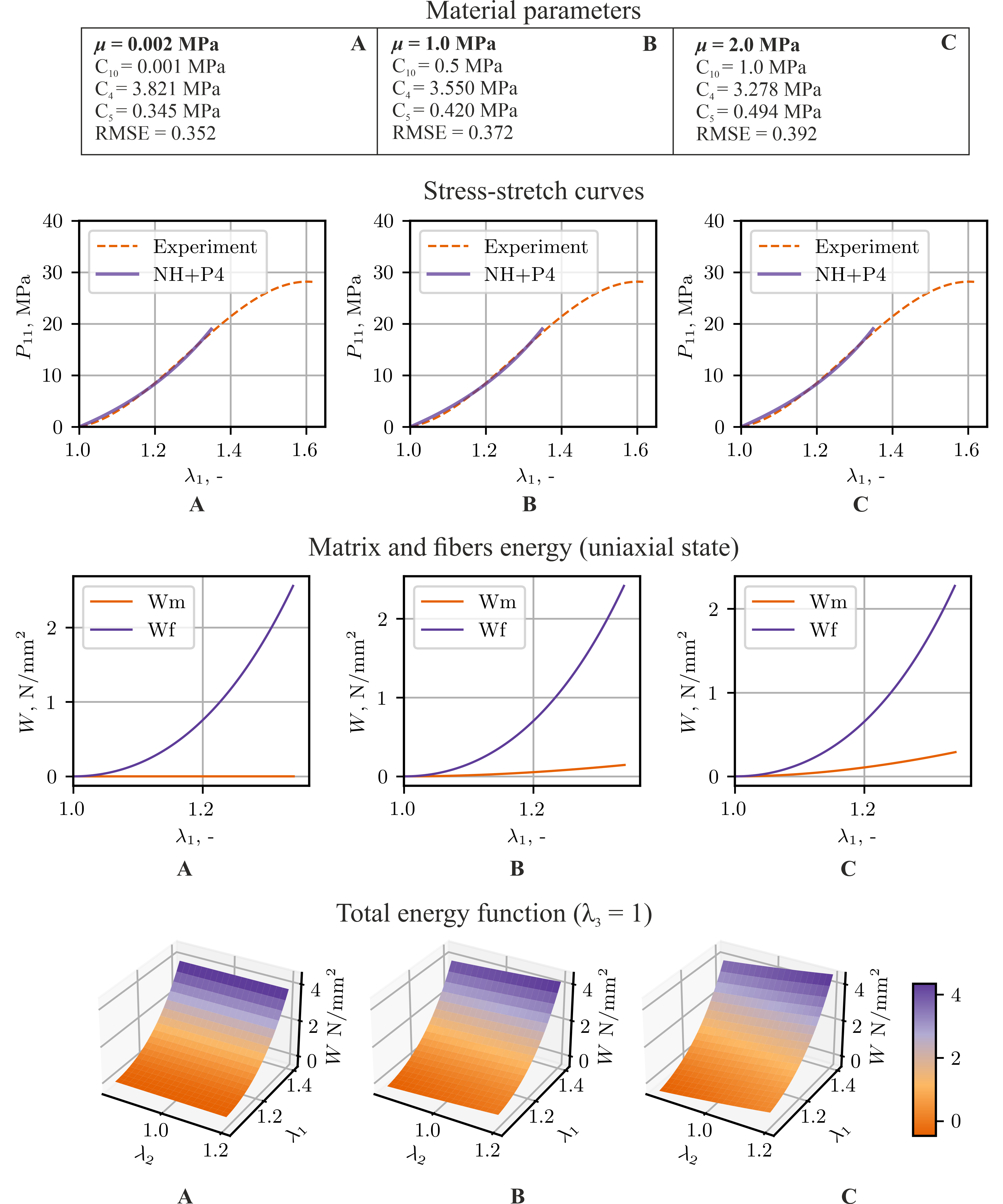


Figure A1. Results of fitting the uniaxial tensile test for ALL ligament, material model NH+P4


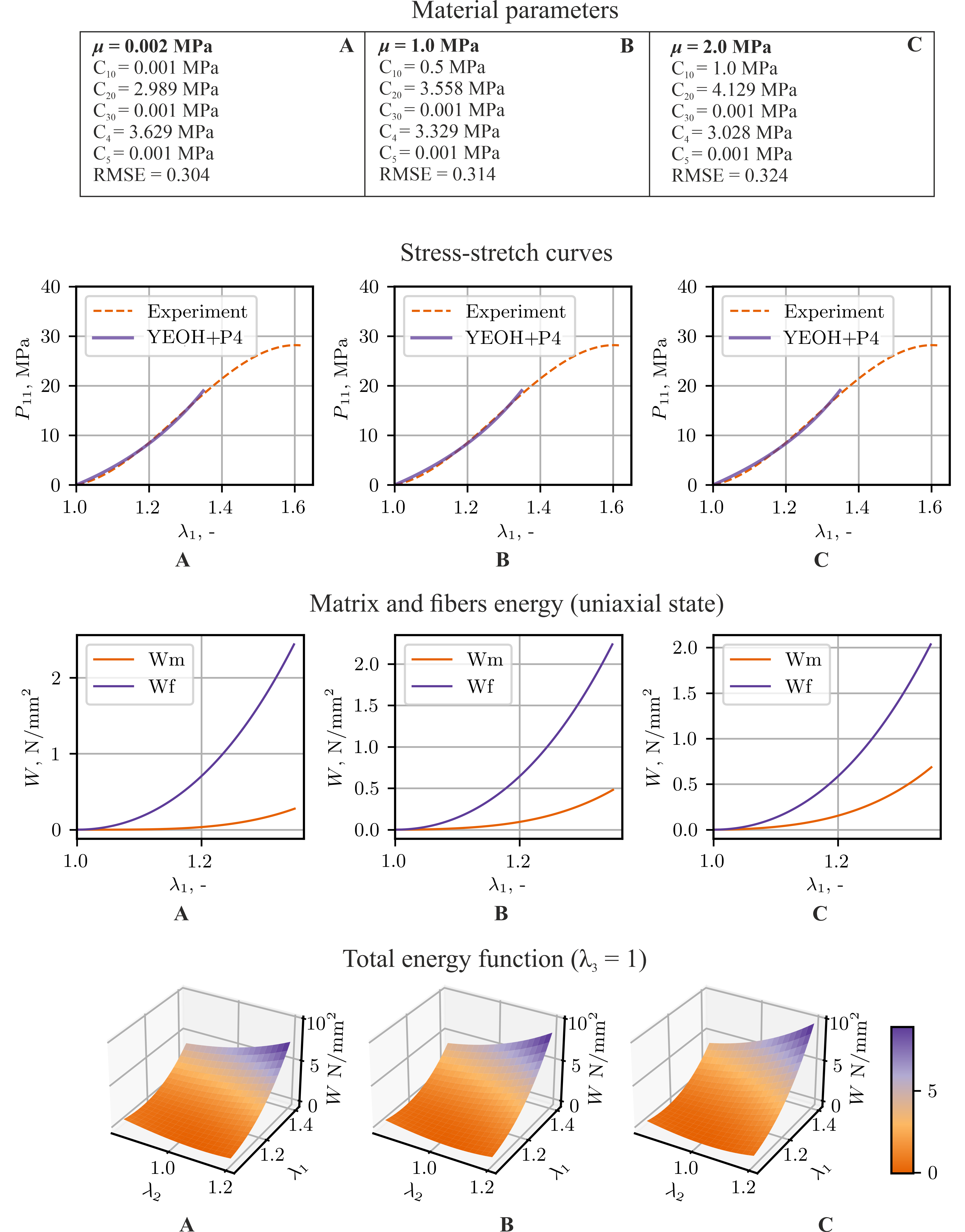


Figure A2. Results of fitting the uniaxial tensile test for ALL ligament, material model Yeoh+P4


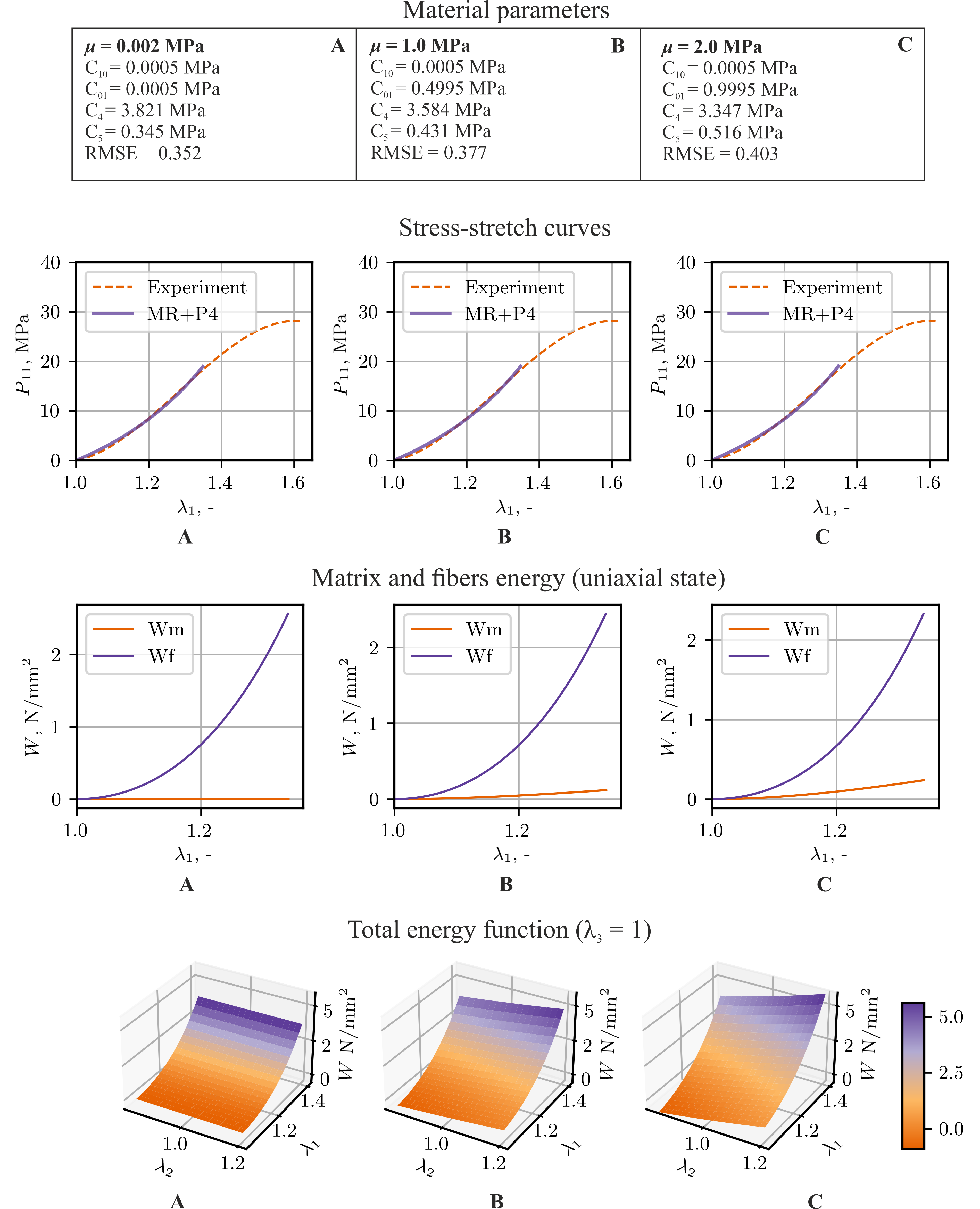


Figure A3. Results of fitting the uniaxial tensile test for ALL ligament, material model MR+P4


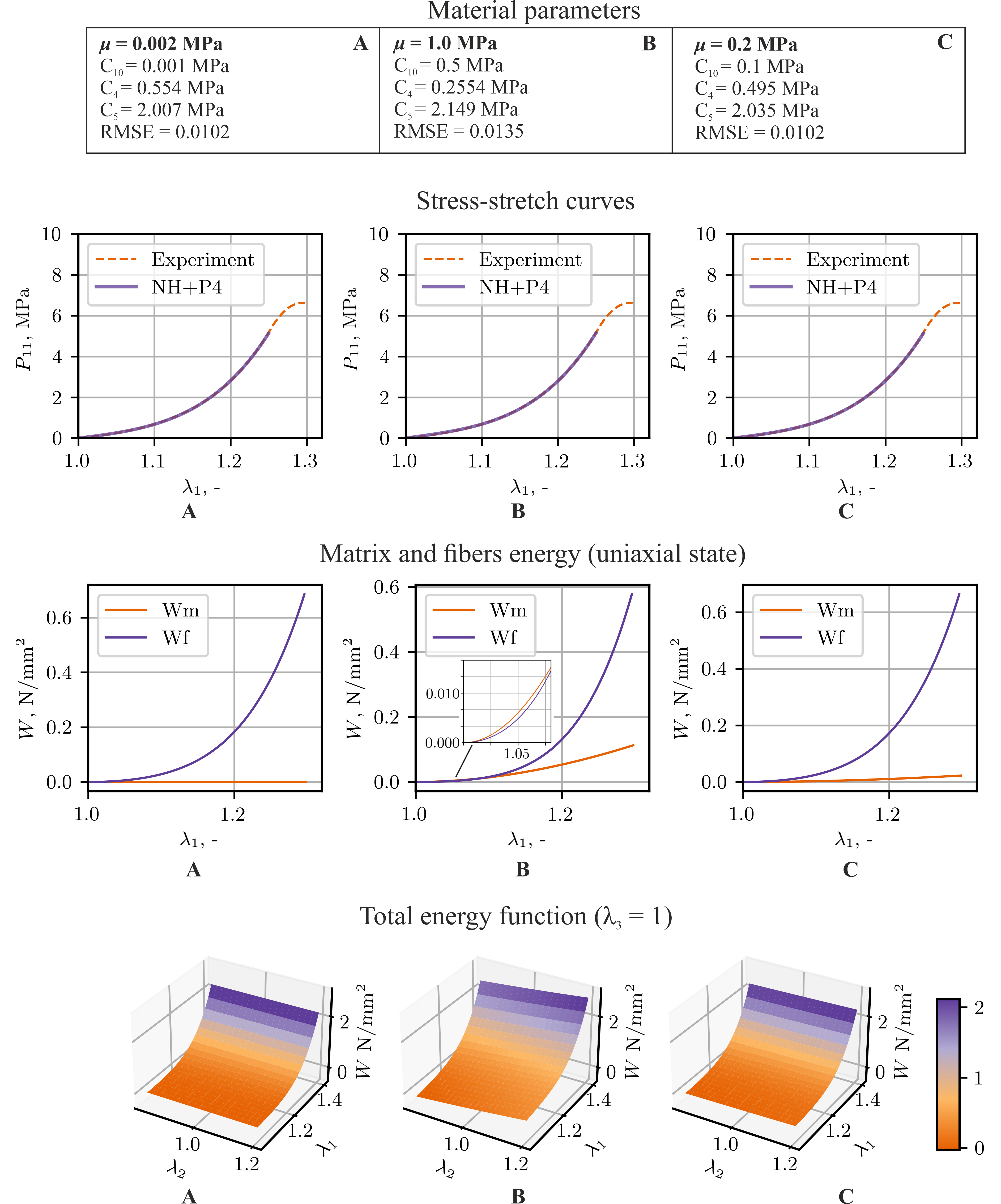


Figure A4. Results of fitting the uniaxial tensile test for LF ligament, material model NH+P4


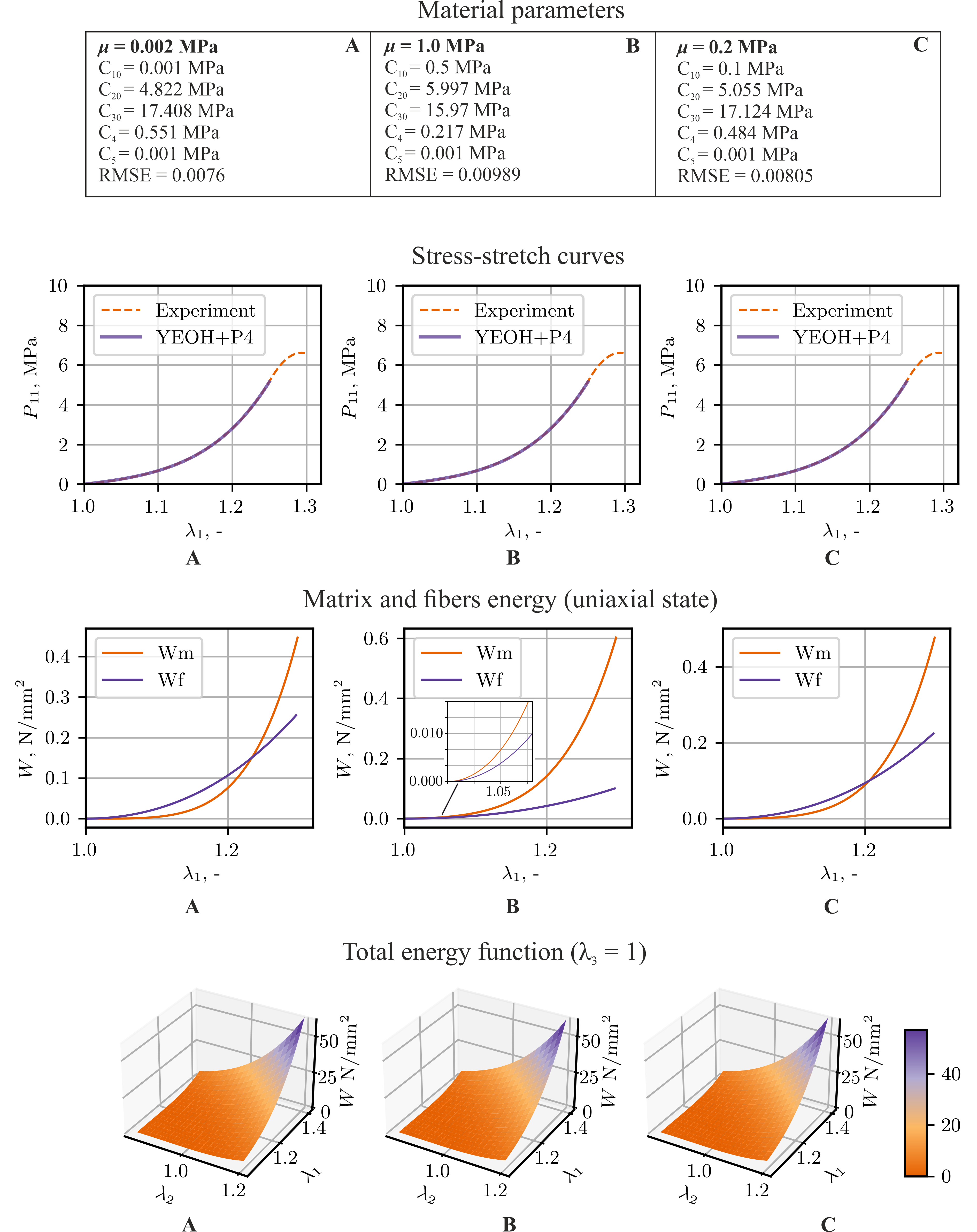


Figure A5. Results of fitting the uniaxial tensile test for LF ligament, material model Yeoh+P4


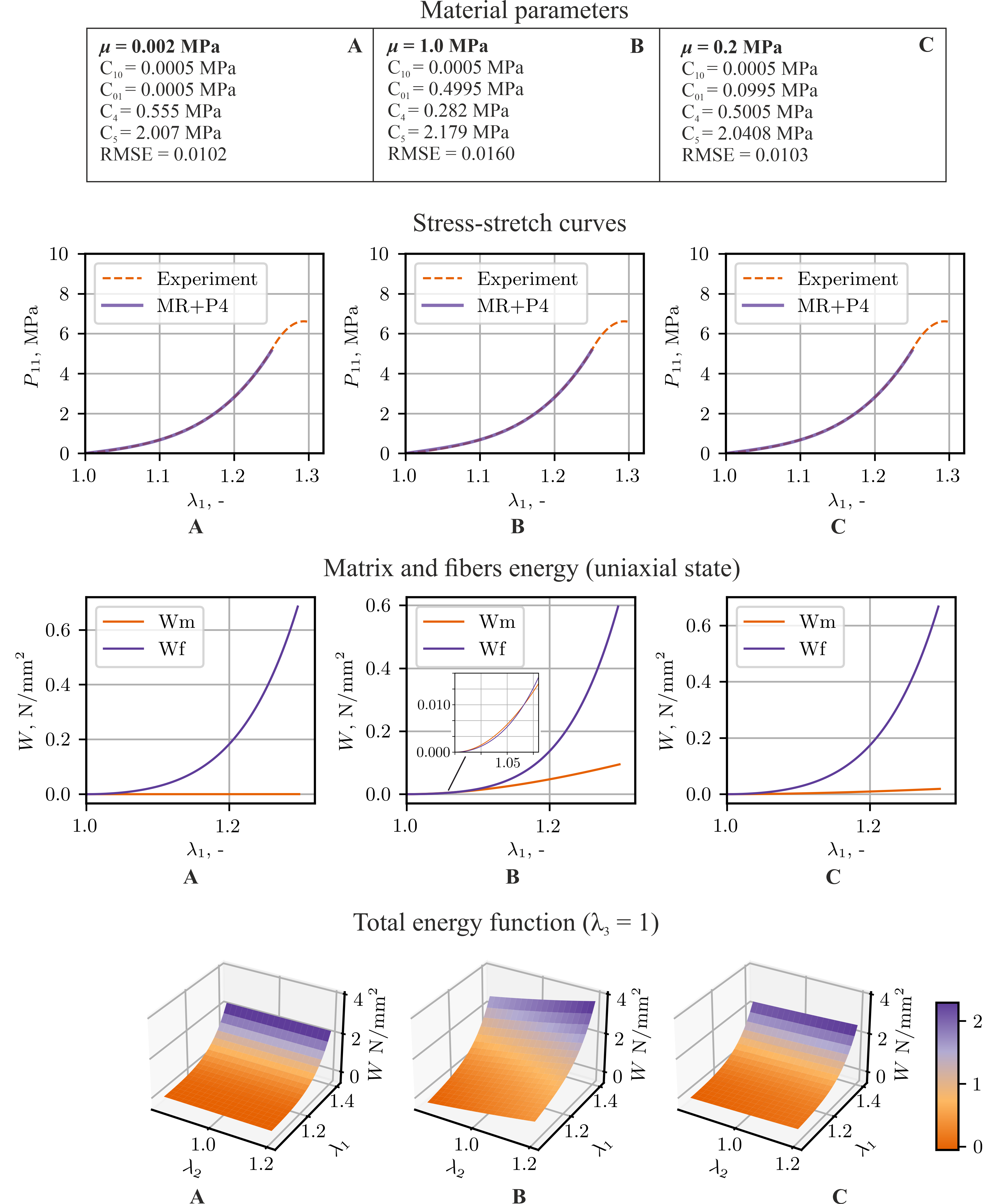


Figure A6. Results of fitting the uniaxial tensile test for LF ligament, material model MR+P4

# Supplementary Material B

This appendix includes the contour maps of axial and transverse stress distribution in soft (LF) and stiff ligament (ALL) obtained with the use of Yeoh+P4 and MR+P4 models in Abaqus.


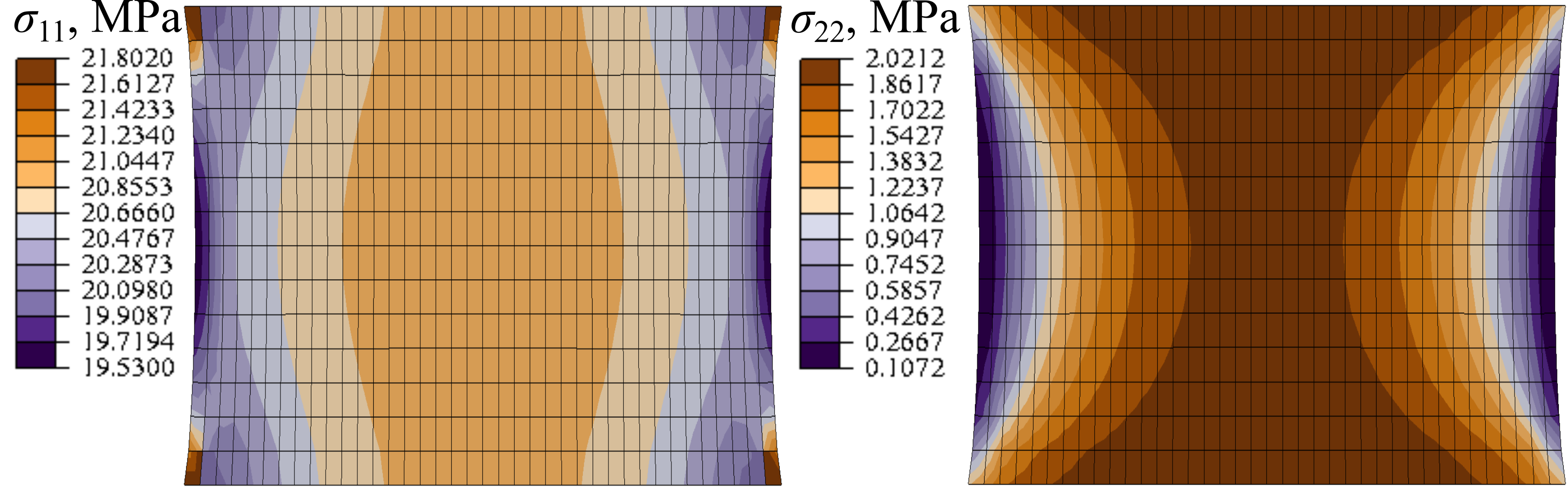


**Figure B1.** Distribution of axial (*σ*_11_) and transverse (*σ*_22_) stress at stretch $\lambda=1.3$; (Yeoh+P4) *μ*=1.0 MPa, ALL, Abaqus


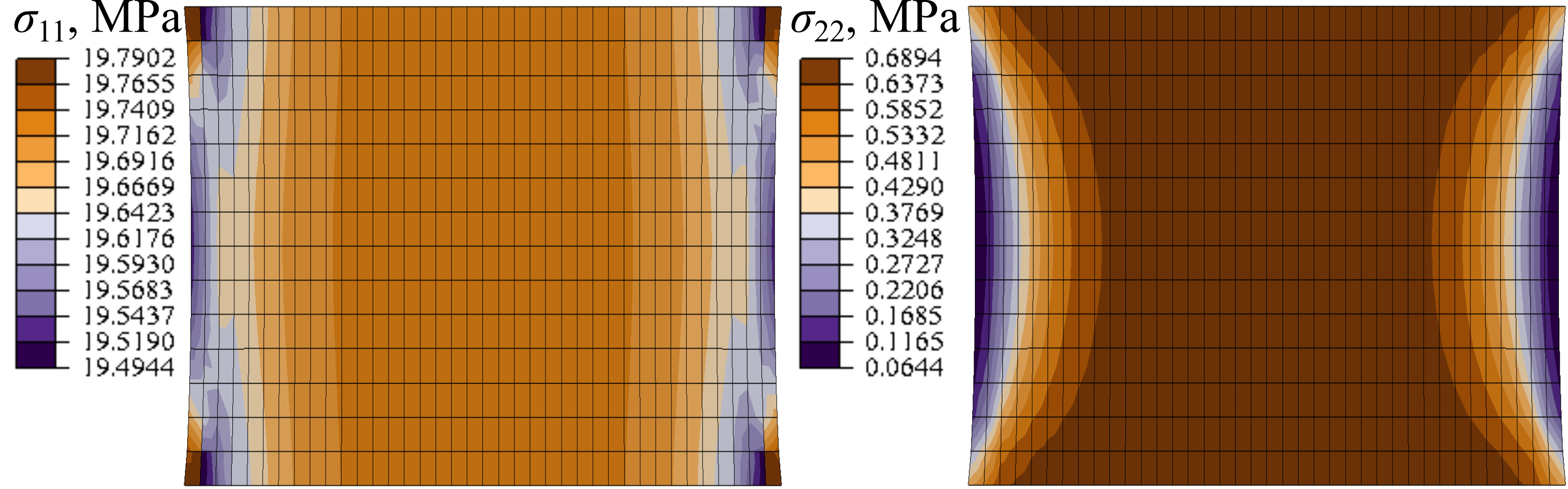


**Figure B2.** Distribution of axial (*σ*_11_) and transverse (*σ*_22_) stress at stretch $\lambda=1.3$; (MR+P4) μ=1.0 MPa, ALL, Abaqus


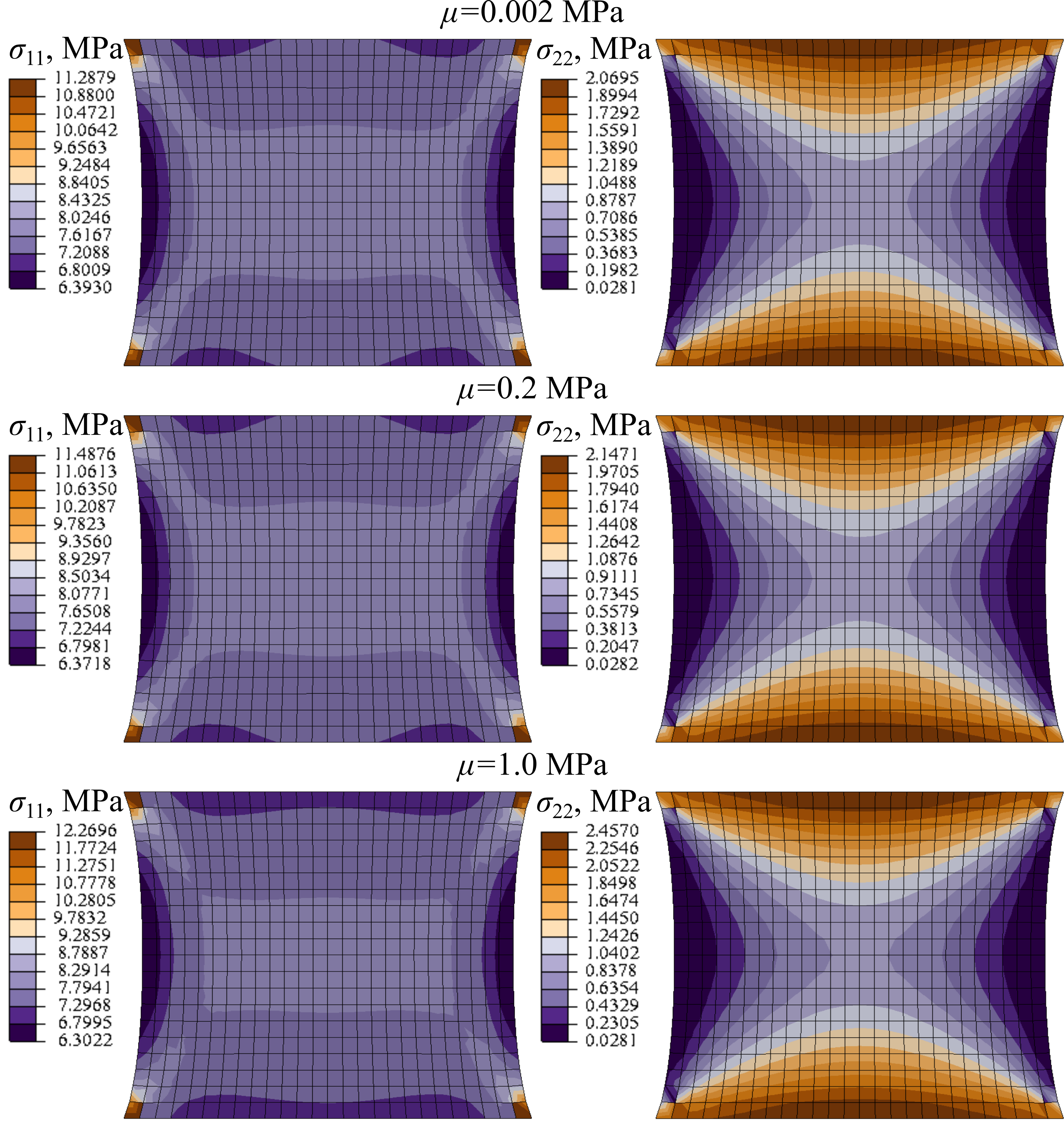


**Figure B3.** Distribution of axial (*σ*_11_) and transverse (*σ*_22_) stress at stretch $\lambda=1.25$ for different material parameters sets (Yeoh+P4), LF, Abaqus

**
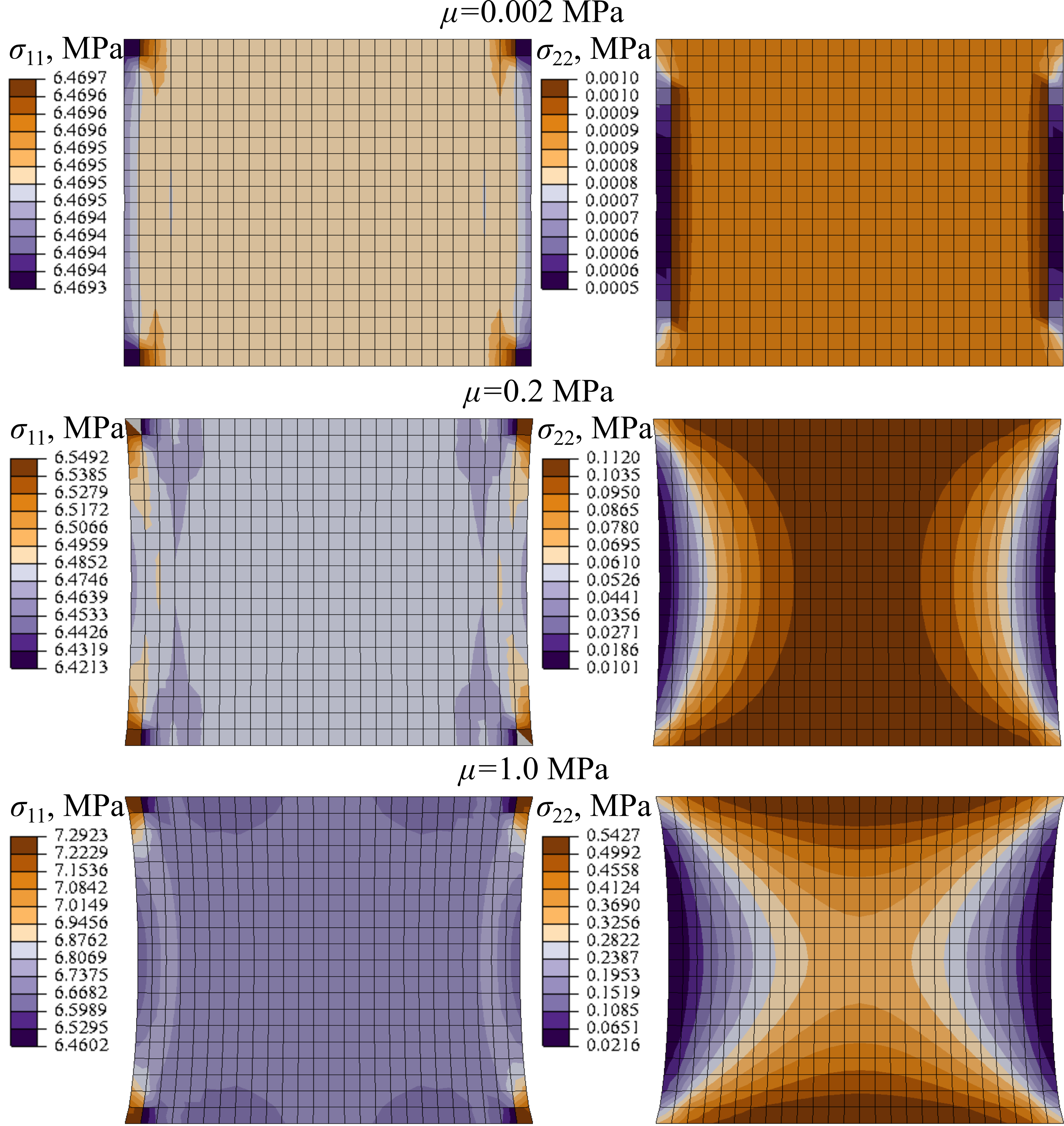
**

**Figure B4.** Distribution of axial (*σ*_11_) and transverse (*σ*_22_) stress at stretch $\lambda=1.25$ for

different material parameters sets (MR+P4), LF, Abaqus
